# Spatially Resolved Mapping of Voltage‐Gated Proton Channel Activity Reveals Delayed Proton Transport in Local Microenvironments

**DOI:** 10.1002/advs.202510837

**Published:** 2025-10-14

**Authors:** Jiahua Zhuang, Yang Xu, Yuxian Lu, Shiyang Lyu, Jie Tan, Jiandong Feng

**Affiliations:** ^1^ Laboratory of Experimental Physical Biology Department of Chemistry Zhejiang University Hangzhou 310058 China; ^2^ Institute of Fundamental and Transdisciplinary Research Zhejiang University Hangzhou 310058 China; ^3^ Hanghzou Institute of Advanced Studies Zhejiang Normal University Hangzhou 311231 China

**Keywords:** cellular electrophysiology, neurodegenerative diseases, proton channels, single‐molecule imaging

## Abstract

Voltage‐gated proton channel Hv1 is a proton‐selective channel that lacks the pore domain typically found in other ion channels, and it is thought to participate in signal transduction by inducing transient, localized changes in proton concentration. However, the contribution of Hv1 channel distribution and activity to the overall pH homeostatic process is still not well understood. Here a single‐molecule photobleaching‐based quantitative imaging method is developed with an Hv1‐fused ratiometric pHluorin probe to study the distribution and dynamics of the proton channel in living bacteria. It is shown that the Hv1 channel does not close sharply when voltage is removed and can directly cause local fluctuations of intracellular pH gradient. Especially, the proton transport through Hv1 is significantly delayed in hypertonic environments, offering novel perspectives at cellular level for neuropathological research. This spatially localized pH microenvironment created by the Hv1 channel may play a crucial role in signal transduction in neuron cells.

## Graphical abstract

A revolutionary imaging platform integrating quantitative single‐molecule photobleaching with ratiometric fluorescence probes enables high‐throughput (≈2300 individually resolvable channels) mapping of individual proton channels in a large field (≈3000 µm^2^), overcoming the spatial and temporal limitations of conventional electrophysiology. This approach has a high sensitivity threshold capable of 7 protons, revealing Hv1 channel kinetics delayed about seven‐folds under pathomimetic hyperosmotic stress.

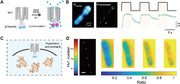

## 1 Introduction

Hydrogen Voltage‐Gated Channel Protein 1 (Hv1) is a membrane protein found in a variety of organisms,^[^ ^1^, ^2^, ^3^ ^]^ responsible for regulating the transmembrane flow of protons. Particularly, the Hv1 channel regulates the pH of the intracellular environment by mediating proton effluxing, thus regulating the packaging, storage, and release of neurotransmitters.^[^ ^4^ ^]^ The measurement of proton currents has advanced our understanding of Hv1's role in brain signaling.^[^ ^1^ ^]^ In neurodegenerative diseases such as Alzheimer's disease (AD) and Parkinson's disease (PD), elevated expression of Hv1 has been shown to correlate with disease severity, potentially exacerbating oxidative stress and neuroinflammation to drive pathogenesis.^[^ ^5^ ^]^ The maintenance of pH homeostasis is believed to support organelles like lysosomes in clearing toxic protein aggregates characteristic of neurodegeneration,^[^ ^6^, ^7^, ^8^ ^]^ suggesting that Hv1‐mediated pH regulation plays a pivotal role in signal transduction, cellular function, and neuropathology.^[^ ^4^ ^]^ However, it remains unclear whether the spatial distribution of Hv1 channels and their proton transport kinetics are altered in local cellular conditions. Investigating the proton transport dynamics of individual Hv1 channels promises insights into the mechanisms by which organisms regulate proton flow to sustain physiological equilibrium and transfer biological information. However, the lack of direct investigation methods to measure the contribution of Hv1 channels to the intracellular pH distribution has raised unanswered questions on the biophysical properties of such proton channels.

Patch clamp technology facilitates direct and immediate observation of single ion channel activity.^[^ ^9^, ^10^, ^11^ ^]^ However, intracellular pH homeostasis is the result of many single channels acting together, rather than acting alone.^[^ ^12^ ^]^ The confined detection area of the patch clamp approach limits its spatial resolution, and by separating the channel locating area from the entire cell during detection, the behavioral details of the channel in the native environment may not be captured.^[^ ^13^ ^]^ Unlike the patch‐clamp method that is based on electrical signals, optical indicators offer a powerful means to map ion channel distribution.^[^ ^14^, ^15^, ^16^, ^17^, ^18^, ^19^, ^20^, ^21^ ^]^ However, correlating the luminescent signal of these indicators with the actual number of channels presents a huge challenge. The susceptibility of fluorescent molecules to photobleaching complicates the direct quantification of single molecules.^[^ ^22^ ^]^ Such obstacles introduce considerable hurdles in the quest to decipher the nuanced behavioral characteristics of individual channels.

Here we develop a single‐molecule photobleaching‐based quantitative imaging approach for revealing proton transport. This platform affords high‐throughput, high‐resolution spatial mapping, and precise quantification, enabling the detailed visualization and localization of the dynamics of proton channels at both whole‐cell and single‐channel levels. Considering that the presence of gated channels in neuronal cells may alter proton flux and confound the detection of Hv1 channel gating dynamics,^[^ ^6^, ^23^, ^24^ ^]^ we performed model validation in Escherischia coli (*E. coli)* that lacks endogenous gated proton channels. To elucidate Hv1 channel behavior within a simplified model that mimics physiological conditions, external voltage was used to polarize the bacterial membrane potential in this Hv1 imaging approach. Our technique afforded direct visualization of proton influx and efflux phenomena with sub‐diffraction limit resolution and directly revealed the contribution of the proton channel in local pH homeostasis that creates a spatially localized pH microenvironment. We observed that, beyond the widely recognized disruption of intracellular pH homeostasis exacerbating oxidative stress and neuroinflammation, prolonged proton equilibration time in Hv1 channels in hypertonic microenvironment induced by toxic protein aggregates may also influence the progression of neurodegenerative diseases.

## 2 Results

### 2.1 Ratiometric Hv1‐pHluorin Probe for Monitoring Whole‐Cell Proton Channel Distribution

**Figure 1:**
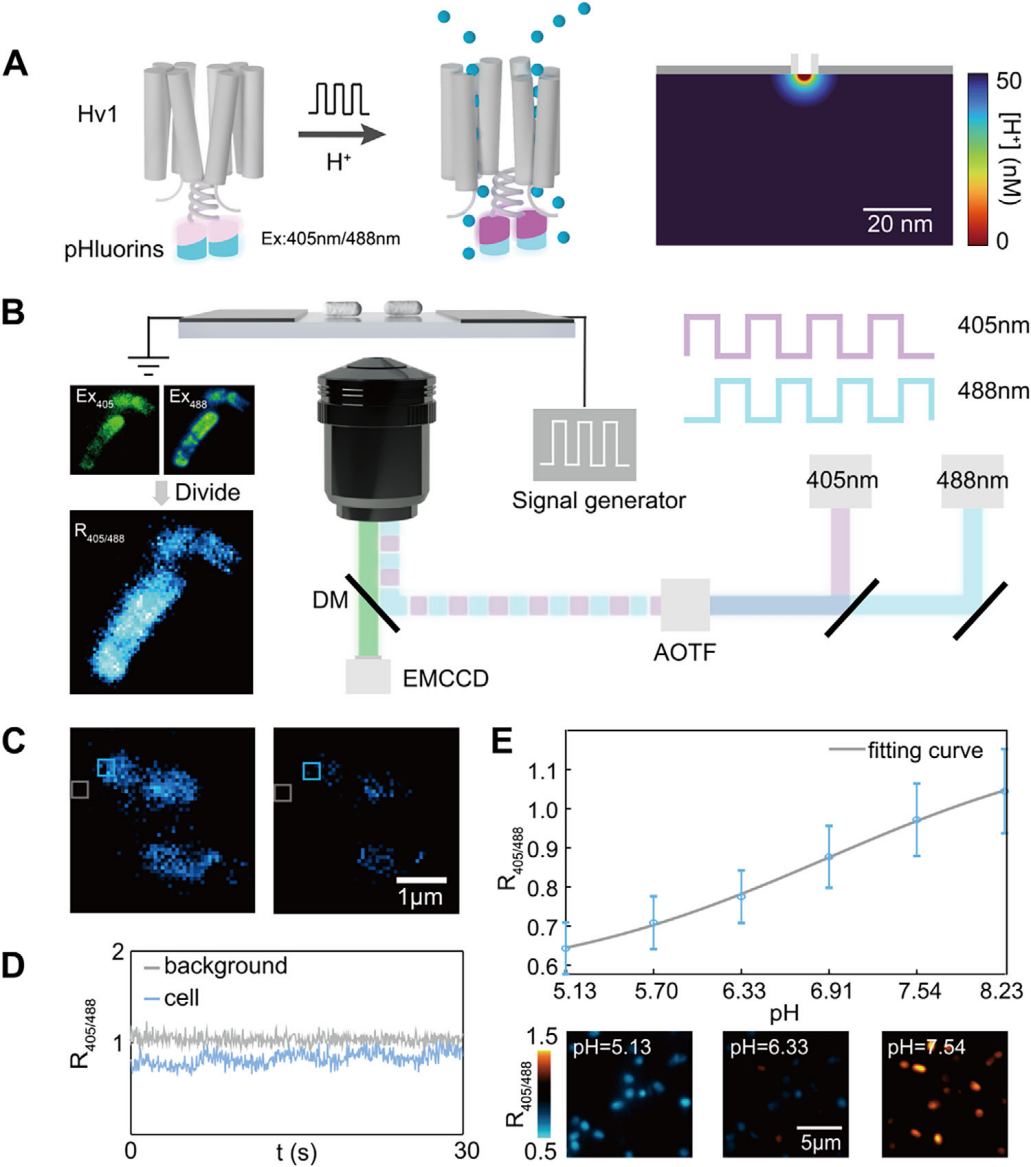
Ratiometric Hv1‐pHluorins probe and the imaging setup. A) Left: illustration of pHluorin‐labeled Hv1 channel opening in response to external electric stimulation; right: FEA result of the distribution of proton concentration in affinity to the open Hv1 channel. B) Illustration of the Experimental setup. The cells are immobilized in the 50‐µm‐interval of the microelectrodes to apply an electrical field. The camera acquisition, the signal generator output, and the stroboscopic laser are synchronized by a DAQ card. The ratiometric image is obtained by calculating the fluorescence intensity ratio between images under 405 nm excitation and images under 488 nm excitation. DM, Dichroic mirror; AOTF, Acoustic Optical Tunable Filters. C) The ratiometric images of Hv1 channels on *E. coli* cells in on/off state (right, left). D) The corresponding ratiometric traces from regions marked by gray box and blue box in C. E) Top: the calibration curve of fluorescence ratio of pHluorins on *E. coli* cells under different pH values acquired using the experimental setup, sensitivity: 7 protons. The data point Bottom: corresponding fluorescence ratio images of pHluorins in *E. coli* cells at pH 5.13, 6.33, and 7.54, respectively. The bacteria were resuspended and observed in phosphate‐citrate buffers with 40 mm benzoate and 10 mm CCCP of different pH. N = 18 cells.

pH‐sensitive fluorescent proteins, whose photophysical properties are modulated by protonation events, serve as exquisite sensors for the quantification of intracellular pH gradients.^[^ ^25^ ^]^ However, besides pH, the fluorescence of these pH‐sensitive proteins can also be affected by factors such as ion concentration and temperature in the microenvironment. Ratiometric (double excitation or double emission) pH sensors partially address these artifacts and enable quantitative pH analysis of cultured cells in a standardized environment. However, due to the incapability of calibrating the local signal, they do not permit the absolute pH value in the microenvironment to be recorded but merely offer a spatially distributed snapshot of the intensity ratio.^[^ ^19^ ^]^ Utilizing protein fusion strategies, in this work we engineered recombinant strains wherein the Hv1 proton channel is fused to a ratiometric pHluorin probe^[^ ^26^ ^]^ at its carboxy terminus, enabling precise measurements of the channel's microenvironmental pH (**Figure** **1**A, left). Laser scanning confocal microscopy images and electrophoresis analysis (Figure S1A,B, Supporting Information) demonstrated that pHluorins were successfully coupled to the Hv1 channel as dimers on the cell membrane of *E. coli*. Finite element analysis (FEA) revealed that the microdomain of Hv1 exhibited a characteristic dimension of ≈20 nm, where the local proton concentration descended with the increase of distance to the center (Figure 1A, right). The pHluorins labeled in close proximity to the Hv1 channel are thus suitable for the in situ, spatially resolved monitoring of proton flux. Considering outer membrane proteins are mostly β‐barrel proteins that reach the outer membrane through complex transport mechanism involving periplasmic chaperones and translocons, in which case the exogenous Hv1‐pHluorin lacks specific bounding sites^[^ ^27^, ^28^ ^]^ and non‐fluorescent state of fluorescent protein in the inclusion body,^[^ ^29^ ^]^ Hv1‐pHluorin are most likely to insert in the inner membrane of *E. coli*, which consists with our observation (Figure S2, Supporting Information). Moreover, the simulations show a pH range of 6.0–7.0 (Figure S3A, Supporting Information) under various viscosity (Figure S3B, Supporting Information), which is optimal for the operational range of ratiometric pHluorins that exhibit a reversible shift in fluorescence emission ratio between pH 5.5 and 7.5 (Figure S4, Supporting Information).

To directly monitor proton channel activities in response to electrical stimuli, we designed a custom electrophysiological apparatus that cooperates with the developed probe (Figure 1B). This setup, including a microfabricated multi‐electrode array, a high‐precision signal generator, and a data acquisition system integrated with an inverted fluorescence microscope, allows for the application of electrical stimulations while simultaneously visualizing cellular responses (Figure S5A, Supporting Information). We employed a stroboscopic illumination technique,^[^ ^30^, ^31^, ^32^ ^]^ where 405 and 488 nm lasers alternately illuminated the sample during the exposure of consecutive frames (Figure S5B, Supporting Information). This optical design allows to monitoring of pH alteration of Hv1 channels using photons collected by two excitation wavelengths of the ratiometric pHluorin probe without compromising the localization performances. Activity initialization of Hv1 channels was triggered by an applied voltage to the bacteria, allowing the selective investigation of Hv1‐mediated proton transport. A distinct reduction in fluorescence intensity was observed, corresponding to Hv1‐mediated proton efflux upon the application of an external depolarizing voltage (Figure 1C). Consistent with the biological function of the efflux proton of the Hv1 channel, we did not observe luminal acidification. The real‐time trace of the fluorescence ratio of intracellular pHluorins showed periodic oscillations in response to the applied electrical field (Figure 1D), illustrating the dynamic equilibrium of proton transport through Hv1 channels. Using Hv1‐pHluorins probes in *E. coli* cells, we established a calibration curve of intracellular pH at a wide dynamic range (5.0–8.0) of the Hv1‐pHluorins probe (Figure 1E), indicating the capability of this method to observe the voltage‐gated Hv1 channel activity in individual *E. coli* cells. We note that due to the heterogeneity of local environments of Hv1‐pHluorins, the fluorescence response to pH change varies, as shown in Figure 1E, and to transfer the fluorescence ratio to the exact pH value is still a goal being pursued. Nevertheless, we can still estimate the sensitivity of our method using the obtained calibration curve as the fluorescence response of different Hv1‐pHluorins are of the same magnitude. Suppose the lowest distinguishable signal intensity is five times of the noise level, the sensitivity of Hv1‐pHluorin is 7 protons.

### 2.2 Hv1 Response to Voltage Stimuli Induced Membrane Depolarization

**Figure 2:**
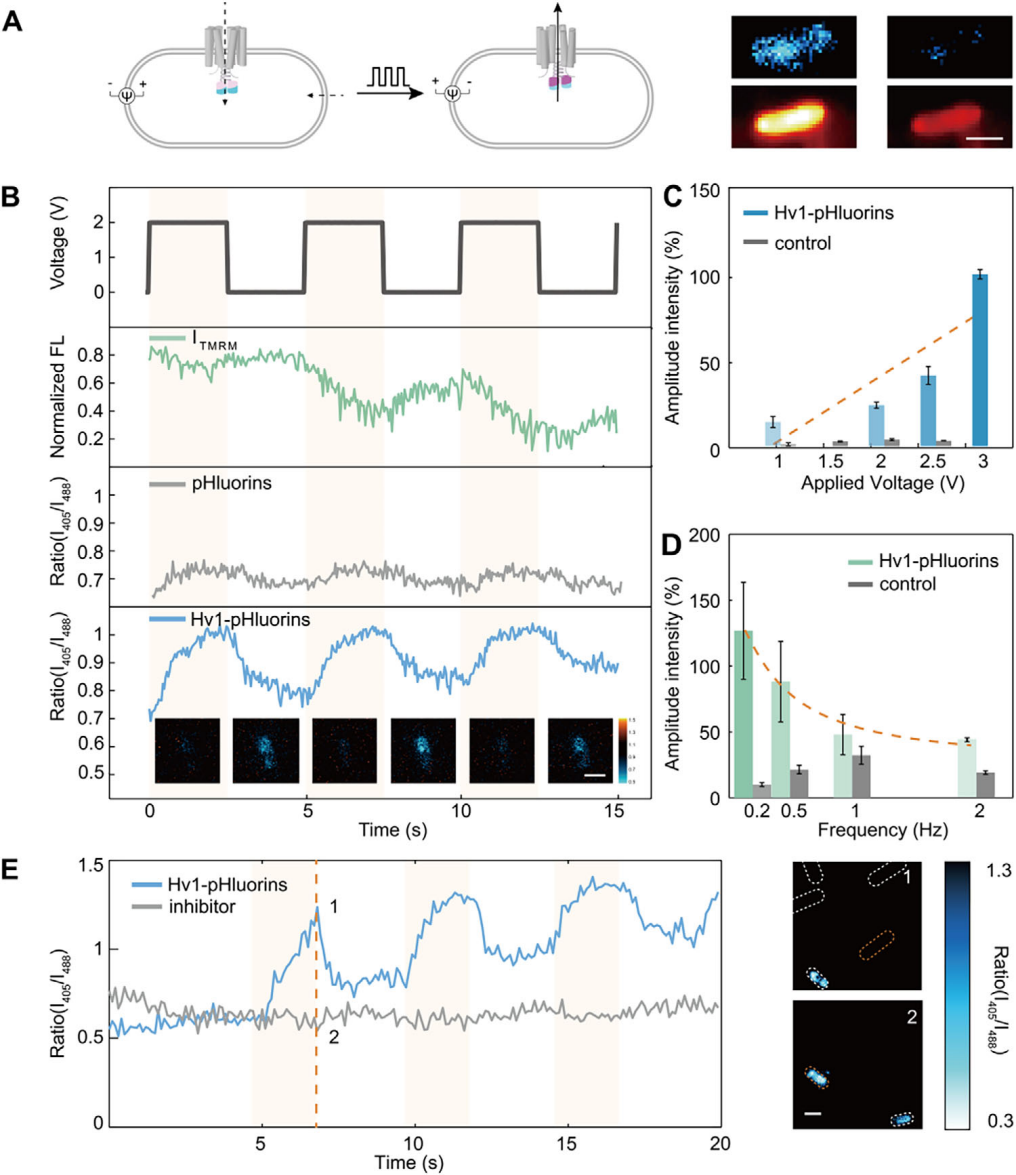
Hv1 channel activity under voltage regulation. A) Left: schematic diagram of the mechanism of Hv1 opening in response to membrane depolarization. Right: the fluorescence ratio images (upper) and the TMRM images (lower) of *E. coli* cells in resting state (left) and depolarized state (right). Scale bar: 1 µm. B) Top: traces of externally applied voltage, normalized fluorescence intensity of TMRM in *E. coli*, fluorescence ratio of pHluorins in *E. coli*, and the fluorescence ratio of Hv1‐pHluorins in *E. coli*. Bottom: Corresponding fluorescence ratio images of Hv1‐pHluorins in *E. coli*. Scale bar: 1 µm. C) The amplitudes of fluorescence ratio fluctuation under different values of applied voltages (1.00–3.00 V, 0.2 Hz) were fitted to the Schwan equation (orange dashed line, R2 = 0.72, RMSE = 34.49). D) The amplitudes of fluorescence ratio fluctuation under different frequencies of applied voltages (0.2–2 Hz, 2 V) were fitted to the Schwan equation (orange dashed line, R2 = 0.98, RMSE = 10.11). E) Left: fluorescence ratio trace of Hv1‐pHluorins in *E. coli* before and after processing by the inhibitor (2GBI); Right: fluorescence ratio images corresponding to the data marked by the orange dashed line in the traces on the left. Scale bar: 1 µm.

Hv1 channels operate in response to depolarization of the membrane potential and transport protons,^[^ ^33^ ^]^ which is postulated to be gated by the membrane potential. We applied periodic pulsed voltages to stimulate cells expressing Hv1‐pHluorins. Concurrent with the application of voltage, we observed a synchronous fluctuation in the fluorescence ratio and the membrane potential indicator tetramethyl rhodamine (TMRM)^[^ ^34^ ^]^(**Figure** **2**A), indicating that the applied voltage altered the induced transmembrane voltage (ITV) on the cellular membrane and thus membrane potential,^[^ ^35^ ^]^ thereby controlling Hv1 channel gating. The ratiometric fluorescence signal of the Hv1‐pHluorin probe thus reflected the kinetic process of the voltage‐induced channel activation upon depolarization.^[^ ^36^ ^]^ The delayed optical response to the voltage pulse demonstrates the slower kinetics of the current‐associated increase in fluorescence, which reflects the change of intracellular pH (Figure 2B, Figure S6, and Movie S1, Supporting Information). Since intracellular pH homeostasis is inherently maintained, this delayed response may result from antagonistic effects of channel closure and intracellular proton supplement.

To examine the impact of the applied voltage and frequency on the electrophysiological regulation of Hv1, we conducted a series of experiments at varying frequencies and voltages and observed systematic alterations in the fluorescence ratio amplitude in response to voltage and frequency (Figure 2C,D). The data were fitted to the Schwan equation: ΔΨ_max_ = 1.5aE(1 + (2πf τ)^2^)^−1/^ ^2^, where ΔΨ_max_ is the induced membrane potential, a is the cell radius, E is the electric field strength, f is the frequency of the alternating electric field, and τ is the membrane relaxation time.^[^ ^37^ ^]^ The fitting results exhibited robust agreement and revealed how the transmembrane potential varies with different electric field intensities and frequencies, providing a foundational basis for studying the cell membrane's response to various electric field modulations. Based on this equation, the induced membrane potential change is calculated to be 37.5–375 mV according to the applied voltage, which is sufficient to regulate the state of Hv1 channels. To substantiate that the voltage‐dependent fluctuations in fluorescence were due to proton dissipation and accumulation via Hv1, 2‐guanidinobenzimidazole (2GBI) was used to inhibit the activity of the voltage‐gated proton channel Hv1.^[^ ^38^, ^39^ ^]^ The addition of 2GBI abolished the periodic fluorescence changes, confirming that the periodic fluorescence ratio signals originate from the proton channel activity (Figure 2E), rather than possible artifacts such as electroporation. These findings demonstrate that the Hv1‐pHluorin probe facilitates the monitoring of the dynamic transition between active and inactive states of Hv1 channels and reveal a heterogeneity of alkalinization rates and pH values among different bacteria. This heterogeneity remains masked in the ensemble averages due to the lack of direct quantification methods to measure Hv1 channels at the single‐channel level.

### 2.3 Single‐Molecule‐Photobleaching‐Based Quantitative Imaging of Hv1 Activity

Although we have developed an imaging apparatus capable of mapping the activity of Hv1 proton channels, the accurate enumeration of these channels remains a formidable challenge. Fluorescent probes are inherently subject to the process of photobleaching, wherein each incident event corresponds to a decay in the fluorescence signal intensity.^[^ ^14^ ^]^ This phenomenon can be harnessed as a proxy for the photophysical activation efficiency of a single fluorophore, thereby offering a molecular counting metric for protein quantification. Capitalizing on this principle, we have innovated a quantitative imaging protocol for revealing proton flux dynamics.

**Figure 3:**
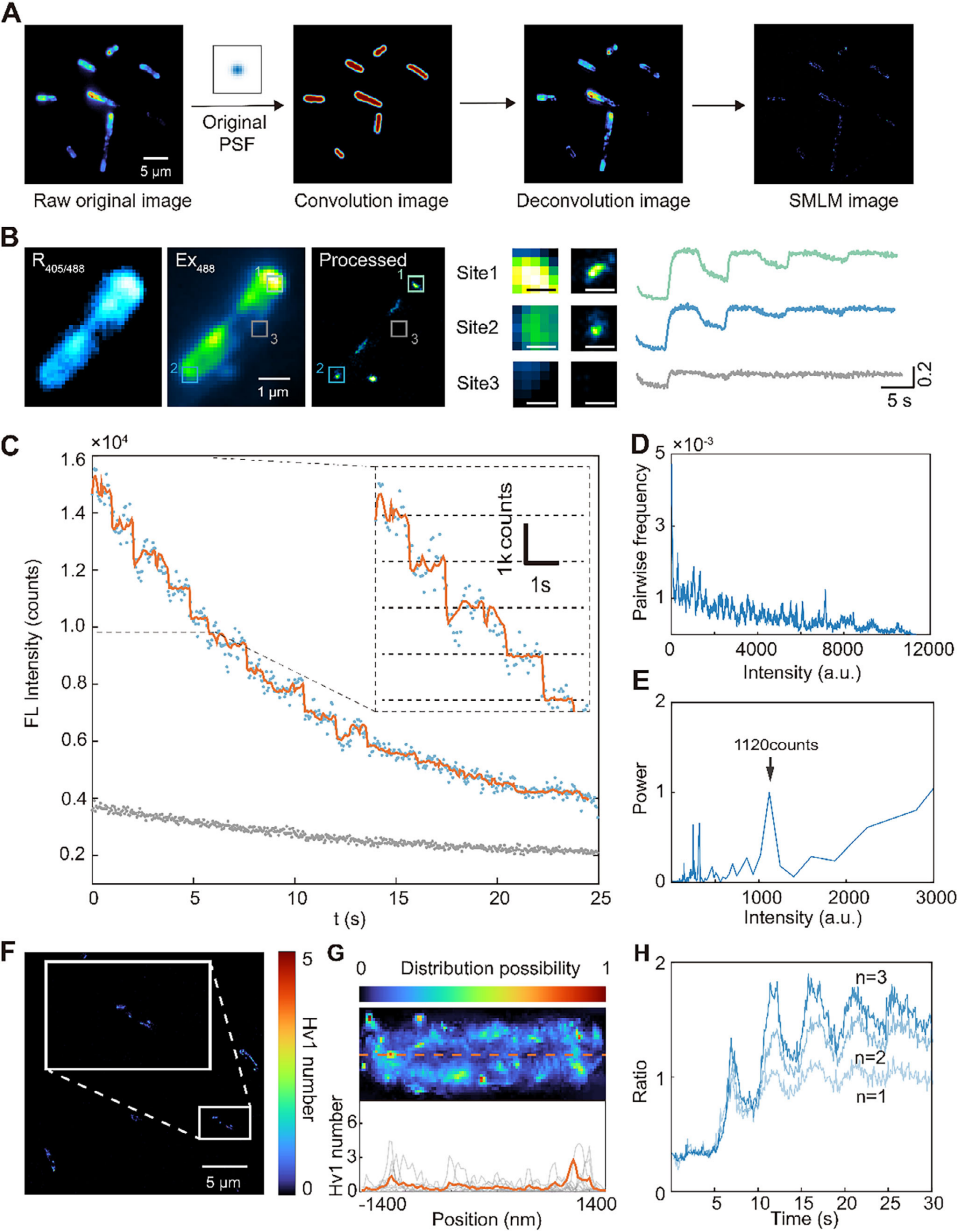
Stepwise photobleaching and single‐molecule localization to analyze Hv1 channel distribution. A) Image processing flow: i) calculating the convolution image of the cell contours and the system PSF, ii) calibrating the fluorescence intensity using the convolution image; iii) applying single‐molecule localization algorithm to determine the localization of Hv1‐pHluorins in cells. B) Left: The result of single‐molecule localization shows distinct localization clusters of Hv1‐pHluorins (R405/488: the ratiometric image; Ex488: the raw image obtained by 488 nm; Processed: the single‐molecule image). Middle: zoomed fluorescence images and single‐molecule localized images of three sites marked with boxes in the images on the left. Scale bar: 100 nm. Right: corresponding traces of fluorescence ratio of the three sites show different behaviors in response to the stimulation of the external electric field. C) The stepwise photobleaching trace of an Hv1‐pHluorin cluster. The zoomed trace in the inset shows the equal value of each step, indicating the observation single‐molecule. D) The pairwise difference distribution spectrum (PDDS) of the filtered trace in B. E) The power density spectrum of PDDS in D shows a peak at 1120, corresponding to the fluorescence intensity of single Hv1‐pHluorins. F) The quantitative single‐channel image of Hv1‐pHluorin. The number of resolved Hv1‐pHluorins is ≈33. G) Top: the statistical distribution probability image of Hv1‐pHluorins in *E. coli*. Bottom: The distribution profile of Hv1‐pHluorins in *E. coli* cells along the orange dashed line. The orange bold line marked the averaged distribution of Hv1‐pHluorins. H) The traces of fluorescence ratio reflect the fluctuations of local protons in near sites with different numbers of Hv1‐pHluorins.

We first located the channel positions using fluorescence images and applied single‐molecule localization algorithms to obtain discrete clusters of channel positions (**Figure** **3**A). The extracted bacterial outlines were convolved with the point spread function (PSF) (Figure S7A–C, Supporting Information) of the system to reconfiguration the Hv1 channel distribution.^[^ ^40^ ^]^ However, in scenarios where multiple channels are in close proximity, forming oligomers or situated within the diffraction‐limited spatial domain, they are optically indistinguishable and can manifest as singular fluorescent entities. The single‐molecule localization algorithm was used to achieve precise spatial mapping and photon counting of each fluorescent punctum,^[^ ^41^ ^]^ thereby promoting spatial localization of Hv1 channel distribution. Continuous monitoring of these channels via time‐lapse imaging elucidated a heterogeneity in response sensitivities to the applied voltage across various localization clusters (Figure 3B).

Due to the optical diffraction limit, the system cannot distinguish between the spots from individual channels or ascribed to clustered channels. Inspired by photoactivated localization microscopy (PALM), we activated multiple fluorescent proteins within putatively multi‐channel clusters and induced photobleaching. The ratio imaging map showed that the pH distribution across the cell membrane was almost uniform, suggesting consistent photoluminescence efficacy of the pHluorins (Figure 3B, left). The irreversible photobleaching of pHluorins allows quantification of the Hv1 channel through the sequential changes in photobleaching curves. The resultant data displayed a stepwise reduction in spot intensity due to photobleaching, with decay steps approximately corresponding to the unit spacing of pHluorins, being consistent with the photobleaching kinetics of single pHluorins molecules (Figure 3C; Figure S7D and Movie S2, Supporting Information). Power spectrum analysis^[^ ^42^, ^43^, ^44^ ^]^ of the pair differential distribution revealed an average step size of ≈1120 ± 100 counts for photobleaching events of single pHluorins molecules (Figure 3D,E). With the quantification of pHluorin molecules, we mapped the distribution of Hv1 channels on the bacterial membrane (Figure 3F) with sub‐diffraction limit resolution (Figure S8, Supporting Information). Statistical mapping of Hv1 distribution on *E. coli* reveals a pattern that aligns with the morphological boundaries of the bacteria, with significant clustering in polar regions (Figure 3G). This distribution characteristic may be related to the function of proton channels in environmental sensing and the adaptation to extracellular fluctuations. In the traces of quantitative proton transport data, we observed doubled or tripled response intensity at one spot, which represents a feature of single‐channel superposition (Figure 3H).^[^ ^15^, ^33^ ^]^ The various response intensity consists well with the photobleaching‐based quantification results and the signal decrease of multi‐channel spot has been interpreted to demonstrate that the activity of multiple single channels can be discretely resolved.

### 2.4 Revealing Opening and Closing Dynamics of Hv1 Channel

**Figure 4:**
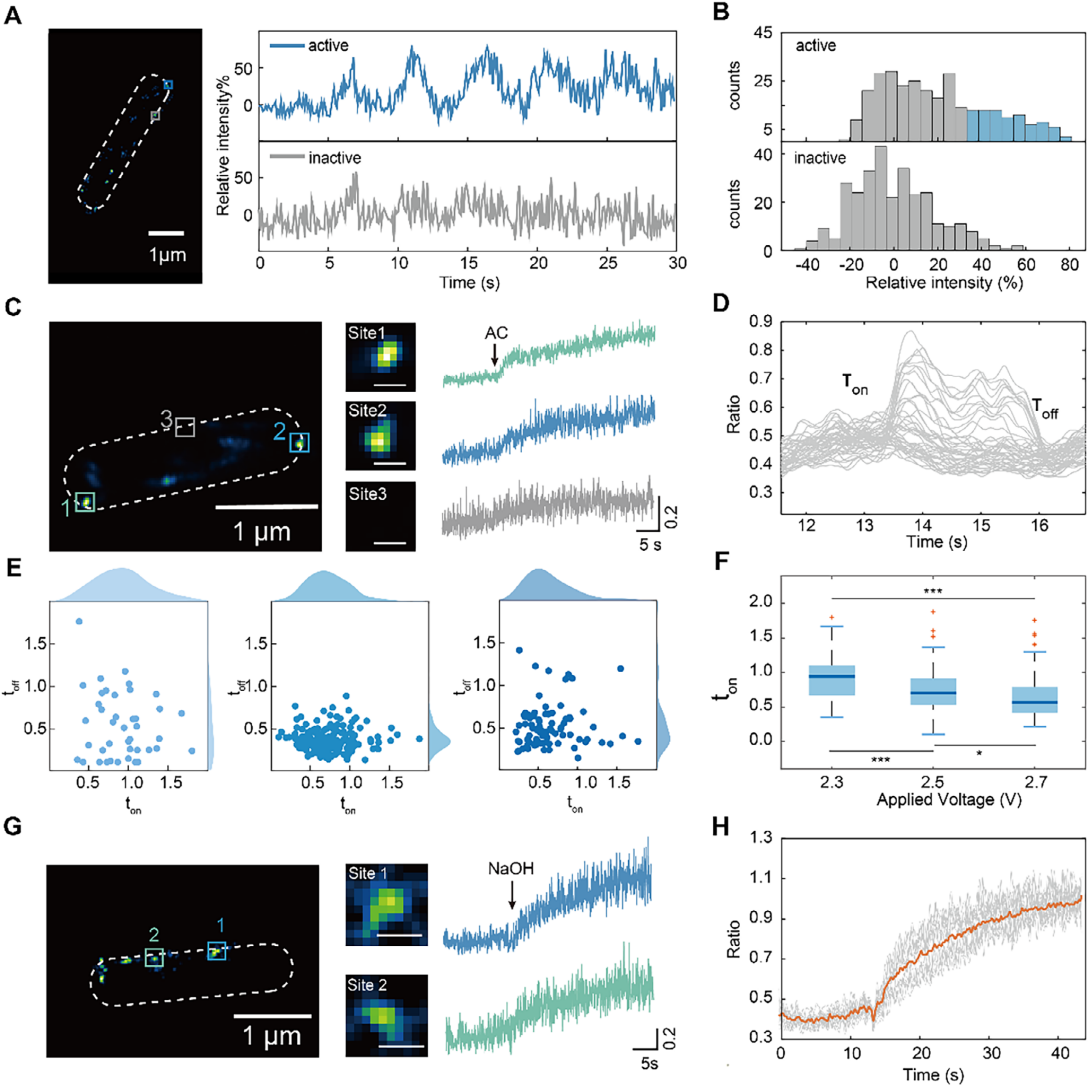
Regulation of Hv1 channel kinetics by voltage gating and pH gradients. A) Left: the quantitative single‐channel image of an *E. coli* cell. The white dashed line marks the cellular outline based on the bright‐field image. Right: traces of normalized change in fluorescence ratios corresponding to the region marked with a blue box and a gray box on the left B) Histograms of traces of the “active zone” (blue) and “inactive zone” (gray) in A, respectively. The difference between the two histograms is marked in blue. C) Left: the quantitative single‐channel image of an *E. coli* cell. Middle: zoomed single‐channel images of three sites marked with boxes on the left. Scale bar: 100 nm. Right: corresponding fluorescence ratio traces of the three sites in response to the stimulation of AC electric field. D) The opening and closing event of different single channels, the activation/deactivation time constants is obtained by the single exponential fitting. E) The scatter plot and the histogram show the fitting results of ton and toff at different applied voltages. F) Box plot of extracted activation time constants under different applied voltages. The significance is shown as ^*^ for *p* ≤ 0.1, ^**^ for *p* ≤ 0.05, ^***^ for *p* ≤ 0.001, ns for no significance.G) Left: the quantitative single‐channel image of an *E. coli* cell. Middle: zoomed single‐channel images of two sites marked with boxes on the left. Scale bar: 100 nm. Right: corresponding fluorescence ratio traces of the two sites in response to the stimulation of NaOH. H) The fluorescence ratio traces of single Hv1‐pHluorins in an *E. coli* cell showed activation kinetics in response to the change of pH gradient. The orange trace is the average of ≈30 single‐channel traces.

Next, we probed the proton transport dynamics within individual channels. Quantitative imaging map showed spatially heterogeneous activation patterns of Hv1 channels, characterized by distinct “active” and “inactive” regions within the cell membrane (**Figure** **4**A). The histogram further indicates that fluctuations in the light intensity of individual spots within these “active” regions reflects the proton channel activity (Figure 4B). Next, we employed photobleaching to reduce the fluorescence to a level where individual channel activity could be monitored over time (Figure S7E, Supporting Information). It was observed that the ratio of fluorescence signal undergoes transient shifts upon voltage application, with the rate of change differing across various positions (Figure 4C). To verify whether the delay in the optical response to the voltage pulse observed in Figure 2B is reproduced at the single‐channel level, the kinetics of channel activation during depolarization and inactivation during repolarization were calculated (Figure 4D; Figure S9, Supporting Information). For Hv1 channels, the activation time constants (t_on_) are often several times larger than the deactivation time constants (t_off_) when detected with a patch clamp. While in our methods, t_on_ and t_off_ were comparable. This may be due to the patch clamp measurement within a confined space, which may overshadow the effect of intracellular pH homeostasis on the opening and closing of proton channels. The intracellular pH distribution is considered to be locally heterogeneous.^[^ ^45^ ^]^ In this case, we can speculate that, when a voltage stimulus is applied, the Hv1 channels open and efflux protons to decrease the local proton concentration, the cells will subsequently supply protons through homeostatic regulation. When the voltage stimulus is removed, the physicochemical proton supplement at the proton channels stops in a slower manner, resulting in the membrane potential not immediately returning to its original state, and leading to the slow closing of Hv1 channels. To verify this speculation, we examined the activation dynamics of single channels under various voltage stimuli. As the intensity of the applied electric field was increased, the distribution of time constant data points evolved from a dispersed to a more aggregated pattern (Figure 4E). Statistically, a significant reduction in the activation time constants was observed, and no appreciable alteration in the deactivation time constants (Figure 4F). This further validates that the activation time constant of the Hv1 channel is related to voltage stimuli, while the inactivation time constant is not.^[^ ^33^, ^36^ ^]^ The activity of the Hv1 channel was observed to be regulated by the pH gradients established across the membrane^[^ ^46^, ^47^ ^]^(Figure 4G). Images showed that the responses of pH stimuli were heterogeneous across different channels, being consistent with the results of voltage stimulation (Figure 4H). These heterogeneities of channel activity observed in response to stimulation are possibly due to the variation of local pH that alters the probability of the channel's active state.

### 2.5 Hv1 Activity Alters Intracellular pH Distribution

**Figure 5:**
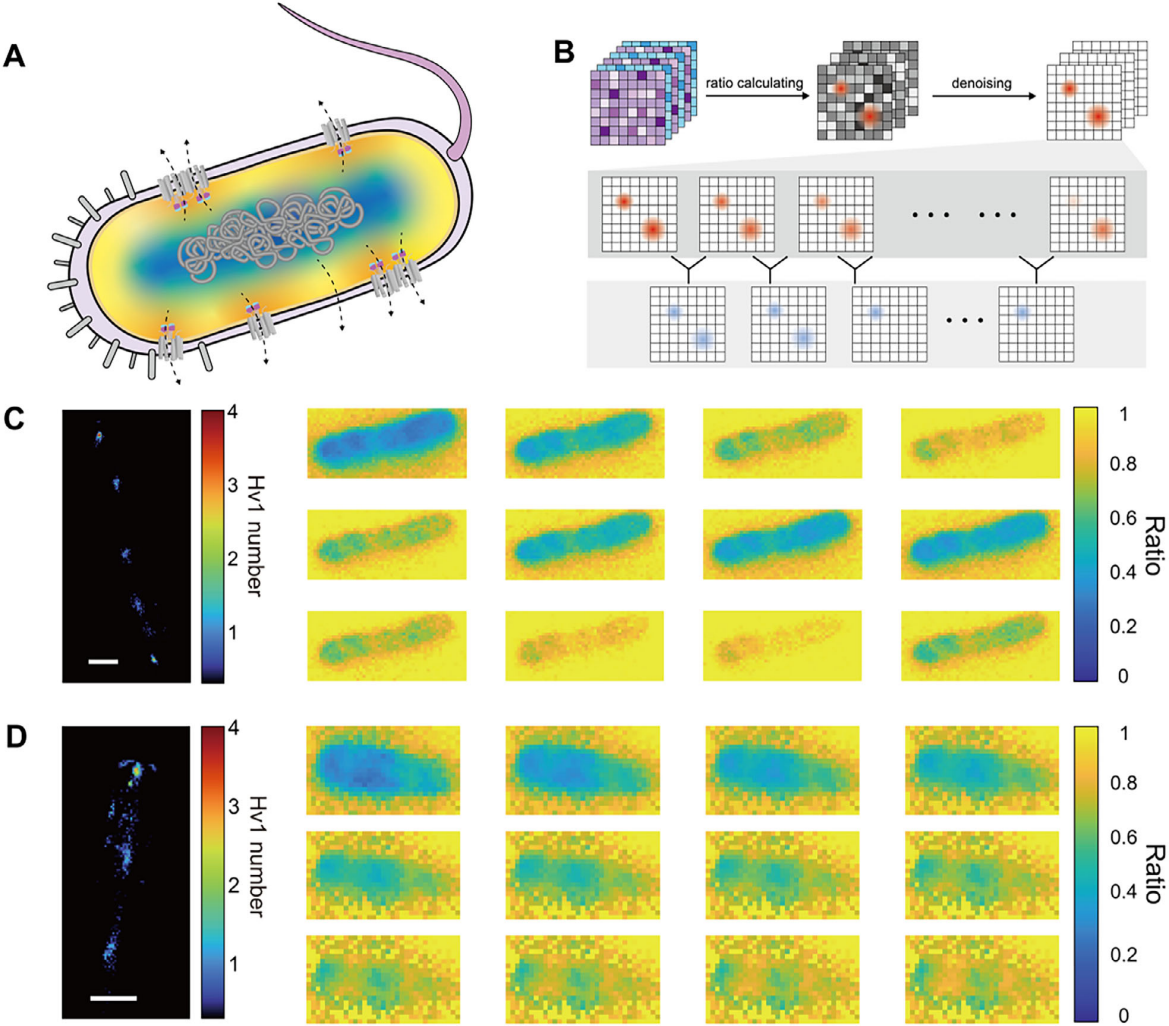
Visualizing active, and inactive states of Hv1 channels and their role in local pH homeostasis. A) Illustration of intracellular proton distribution. B) Data process flow of the spatial‐temporal analysis. Time‐resolved fluorescence ratio mapping (middle row) was obtained by denoising the calculated fluorescence ratio images. The difference between two fluorescence ratio mappings (bottom row) reveals the dynamic parameters like activity and response time of Hv1 channels at different sites. C) Distribution of Hv1‐pHluorins in an *E. coli* cell under repetitive electric field stimulation (2.5 V, 0.2 Hz) (left) and corresponding time‐resolved fluorescence ratio mapping image snapshots (right). Scale bar: 1 µm. D) Distribution of Hv1‐pHluorins in an *E. coli* cell under external pH stimulation (add 0.1 m NaOH in 0.01 m HEPES, pH 7.5) (left) and corresponding time‐resolved fluorescence ratio mapping image snapshots (right). Scale bar: 1 µm.

Theoretically, the above‐mentioned slow response of Hv1 channels and the subsequently induced local pH differences can be observed in the overall pH imaging of the bacteria (**Figure** **5**A). To verify this phenomenon, the image sequence of fluorescence ratio within bacteria is obtained by extracting images at fixed intervals and applying a denoise algorithm (Figure 5B). By calculating the difference of two consecutive fluorescence ratio images in the image sequence pixel by pixel, the image sequence of fluorescence ratio changing rate images was obtained to show the channel activity and the local pH change rate (Figure S10, Supporting Information). This algorithm processing enables visualization of the changes in different channel activity and internal pH values within whole bacteria during different voltage stimulation cycles. It was found that the distribution of activated Hv1 channels was always consistent across cycles, accompanied by the variation of local pH value (Figure 5C). This Hv1 channel‐induced intracellular pH discontinuity may be due to the extremely low rate of physicochemical proton supplement in the cytosol, which is attributed to the transient and reversible association of protons with ionizable groups on cytosolic macromolecules, lengthening the time taken for them to wander in the cytosol.^[^ ^48^, ^49^ ^]^ Surprisingly, in both the voltage‐ and pH‐ stimulation, the intracellular pH distribution was highly similar to the distribution of the most active Hv‐1 channel, displaying a recurring acidic “hot area” in polar regions (Figure 5D). In theory, the activation of Hv‐1 channels represents a large efflux of protons (Figure S11, Supporting Information), these regions are habitually thought to be alkaline. This simplistic view ignores the fact that the alteration of the intracellular pH homeostatic process is delayed. This strange convergence may be due to the aggregation of Hv1 channels at the high curvature of the bacteria, resulting in a rapid decrease of local pH when the channels are opened, and the delayed flow replenishment of intracellular protons induces acidic loci that overlap with the distribution of intracellular Hv‐1 channel clusters. In summary, due to the heterogeneous channel distribution and response properties of proton channels, the overall and local pH in the cell will change differently, and these characteristics may have important effects on cellular physiological activities such as signal transduction within neuronal cells. These pH homeostasis alterations are hypothesized to impact cellular functions such as protein degradation and redox balance, potentially contributing to the pathogenesis of various diseases, including Parkinson's disease(PD), Alzheimer's disease (AD), and Amyotrophic Lateral Sclerosis (ALS).^[^ ^50^, ^51^, ^52^ ^]^ In recent years, the Hv1 channel has emerged as a critical therapeutic target in neurodegenerative diseases.^[^ ^53^, ^54^ ^]^ Modulating Hv1 overactivity may induce neurodegeneration by perturbing intracellular pH homeostasis and cellular function.

### 2.6 Delayed Proton Transport in Hypertonic Environments

**Figure 6:**
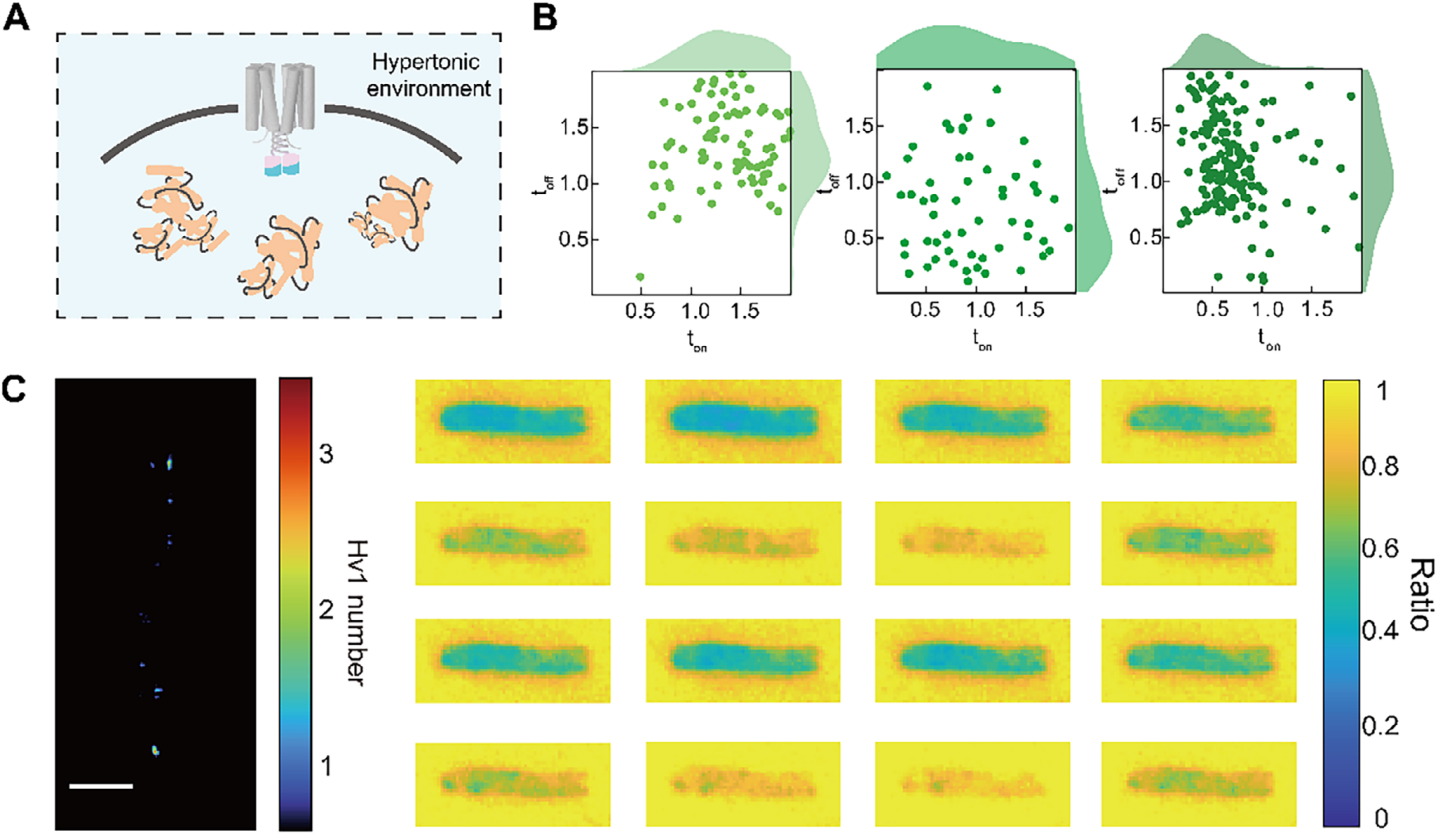
The Hv1 dynamic response in a hypertonic solution environment. A) The schematic diagram of Hv1 in a hypertonic solution environment. B) Scatter plot and histogram illustrating the fitting results of ton and toff at various applied voltages in the hypertonic solution environment. The external solution of the bacteria was replaced by a 100 mm HEPES (pH 7.5) buffer with a sucrose concentration of 20%. C) Distribution of Hv1‐pHluorins within an *E. coli* cell under repetitive electric field stimulation (2.7 V, 0.2 Hz) (left), along with corresponding time‐resolved fluorescence ratio mapping image snapshots (right). Scale bar: 1 µm.

To investigate the effects of proton channel distribution and activity in neurodegeneration, we employed Hv1 channels in live bacteria as a model system. A hallmark pathology of neurodegenerative diseases is the accumulation of cytotoxic protein aggregates,^[^ ^55^ ^]^ which increases cellular osmotic pressure (**Figure** **6**A). We thus use hypertonic environments to mimic protein aggregation by elevating cytoplasmic solute concentration. Under hypertonic conditions, we observed no fundamental changes in channel gating mechanisms (Movie S3, Supporting Information), but the t_on_ and t_off_ significantly increased compared to controls (Figure 6B; Figure S12, Supporting Information). This phenomenon may arise from restricted proton diffusion, which prolongs the regulatory response of local proton concentration gradients on channel activity, leading to reduced proton transport rates. While hypertonic environments indirectly modulate Hv1 channel dynamics by altering cytoplasmic physical properties (e.g., viscosity, proton diffusion), it does not disrupt its voltage dependency. The adaptive slowing of Hv1 kinetics under hypertonic environments may alter the intracellular pH homeostatic process, potentially destabilizing local pH homeostasis. (Figure 6C). These findings provide novel insights into the regulation of membrane protein function under hypertonic environments. However, the specific impact requires further validation in complex in vivo environments. This mechanism may offer new perspectives for studying neurodegenerative pathologies, such as improving neuronal function by modulating cytoplasmic physical states.

## 3 Conclusion

In this study, we have developed a single‐molecule photobleaching‐based quantitative imaging technology that facilitates parallel and real‐time tracking of channel responses to extracellular electric fields and pH gradients, thereby elucidating the distribution, the activity, and the kinetic heterogeneity of Hv1 channels at the single‐molecule level. Unlike patch clamp techniques that detect channel opening and closing in confined spaces, our method can resolve Hv1 channel activity and distribution within the cellular pH homeostatic system near more native states. Spatial information shows that proton channels can not only induce local pH gradients in the cytosol but also cause gradients from one end of the bacterial cell to the other. Such proton gradients within cells may play a key role in driving ATP biosynthesis and the accumulation or extrusion of a variety of solutes to orchestrate cellular metabolism, and in transmitting biological information. Furthermore, we observed that hypertonic environments decelerate the kinetics of Hv1‐mediated pH homeostasis maintenance, offering novel perspectives at cellular level for neuropathological research. By simply changing the fused pHluorin fluorophore to corresponding ion‐sensitive fluorophores, this technique can be easily expanded to other voltage gated channels (e.g., Ca^2+^ channels, K^+^ channels, and Na^+^ channels), facilitating the development of neural science, tumor immunology, cell–cell interaction and communications, and synthetic biology. Through this, our technique will benefit the development of high‐content screening of channel protein‐targeted drugs by providing modulation and extra electric‐field dependent gating information.

## 4 Experimental Section

### Reagents

LB (Luria‐Bertani medium), M9 (M9 Minimal medium, Shanghai yuanye Bio‐Technology Co., Ltd), ampicillin, kanamycin, polylysine, citric acid, K_2_HPO_4_, CCCP (Carbonyl Cyanide m‐Chlorophenylhydrazone), TMRM (Tetramethylrhodamine), SDS (Sodium dodecyl sulfate), KCl, NaCl, HEPES (2‐[4‐(2‐hydroxyethyl)piperazin‐1‐yl]ethanesulfonic acid), β‐mercaptoethanol (Macklin), proteinase inhibitors (MedChemexpress), Lysozyme (Macklin), DNAse1 (Shanghai yuanye Bio‐Technology Co., Ltd), Ni‐NTA agarose (LabLead).

### *E. coli* Strains

A target sequence of pHluorins – the green fluorescent protein variant was engineered and constructed at the C‐terminal of the Hv1 channel and cloned it onto the pET‐28(+) plasmid, which was subsequently transformed into *E. coli* BL21 (DE3). The transformed cells were oscillated overnight at 220 rpm at 37 °C for pre‐culture. And next day the pre‐culture was diluted at a ratio of 1:1000 to fresh M9 medium (2% glycerol, 4% amino acids (V/V), 2% Vitamins (V/V)) with 50 µg mL^−1^ kanamycin at 37 °C to medium logarithm (OD_600nm_ = 0.4–0.5). The expression of Hv1‐pHluorins was induced by adding 0.6 mM IPTG, and the cells were harvested after continued culture at 37 °C for 4 h for further use.

### Amino Acid Sequence of Hv1

MATWDEKAVTRRAKVAPAERMSKFLRHFTVVGDDYHAWNINYKKWENEEEEEEEEQPPPTPVSGEEGRAAAPDVAPAPGPAPRAPLDFRGMLRKLFSSHRFQVIIICLVVLDALLVLAELILDLKIIQPDKNNYAAMVFHYMSITILVFFMMEIIFKLFVFRLEFFHHKFEILDAVVVVVSFILDIVLLFQEHQFEALGLLILLRLWRVARIINGIIISVKTRSERQLLRLKQMNVQLAAKIQHLEFSCSEKEQEIERLNKLLRQHGLLGEVN

### Amino Acid Sequence of P2A Linker

GSGATNFSLLKQAGDVEENPGP

### Amino Acid Sequence of pHluorin

MSKGEELFTGVVPILVELDGDVNGHKFSVSGEGEGDATYGKLTLKFICTTGKLPVPWPTLVTTFSYGVQCFSRYPDHMKRHDFFKSAMPEGYVQERTIFFKDDGNYKTRAEVKFEGDTLVNRIELKGIDFKDDGNILGHKLEYNYNEHLVYIMADKQKNGTKAIFQVHHNIEDGGVQLADHYQQNTPIGDGPVLLPDNHYLHTQSALSKDPNEKRDHMVLLEFVTAAGITHGMDELYK

### Purification of Hv1‐pHluorins

The gene of His‐tagged Hv1‐pHluorins was inserted into the pET‐28a(+) plasmid vector, and the recombinant plasmid was loaded in BL21 (DE3) E.coli.^[^ ^56^ ^]^ The process was done by GenScript Biotech Corp. The transformed cells were grown in 1 L, lysogenic broth medium (LB medium) with 50 µg mL^−1^ kanamycin at 37 °C to an OD 600 of 0.6. Expression of Hv1‐pHluorins was induced by adding 0.8 mM IPTG and continued growing at 20 °C overnight. Cells were harvested by centrifugation (4500 × g, 30 min, 4 °C). The cell pellets were resuspended in 20 mM HEPES, 150 mM NaCl, 5 mM β‐mercaptoethanol, pH 8, and a protease inhibitor cocktail. Lysozyme (320 ug mL^−1^) and DNAse1 (60 µg/mL in 1 m MgCl_2_) were added, and the suspension was stirred at 4 °C for 60–90 min. An ultrasonic cell disruptor was used to break the sample for ≈50 min (2 s of ultrasound followed by 2 s stop). The broken pieces were separated by centrifugation (4500 × g, 20 min, 4 °C), and the supernatant was followed by ultracentrifugation (90 000 × g, 4 °C, 90 min). The resulting membrane precipitation was resuspended in 20 mM HEPES, 150 mM NaCl, 1% SDS (w/v), and pH 8 at room‐temperature. DNAse1 (2.4 mg) was added and stirred for 60 min followed by centrifugation (15 000 × g, 20 °C, 60 min). The remaining supernatant was incubated with Ni‐NTA agarose at room‐temperature overnight. After the resin was rinsed with 3 column volumes of 20 mM HEPES, 150 mM NaCl, 1% SDS, pH 8, the protein was eluted with 20 mM HEPES, 150 mM NaCl, 1% SDS (w/v), 250 mM imidazole, pH 8. An SDS‐PAGE (sodium dodecyl sulfate‐polyacrylamide gel electrophoresis) documented the purity.

### Micro‐Electrode

Glass coverslips (0.17 mm thickness, 25 × 25 mm) were cleaned with acetone, isopropanol, and water before photolithography and magnet sputtering. The micro‐electrode layer consists of 20 nm Ti and 300 nm Pt. The pattern includes three work electrodes for applying an electric field and one counter electrode that serves as ground. The interval between the work electrode and counter electrode varies from 20 to 50 µm as a balance between electric field strength and experiment throughput.

### Cell Electrophysiological Set‐Up

The device consists of a base, a customized PCB (Printed Circuit Board), and a solution chamber. The PCB board was mounted on the base, connecting micro‐electrodes through metal clips and a signal generator through coaxial interface. The solution chamber is mounted on the PCB board to hold buffers and mediums for cell culture.

### Fluorescence Imaging of Live *E. coli* Cells

*E. coli* cells cultured in 37 °C M9 medium were harvested by centrifugation. The harvested cells were fixed on a 0.01% PLL (Poly‐L‐Lysine)‐modified cover slide, and the unfixed cells were washed away sufficiently by M9 medium. All experiments were conducted at room‐temperature (22–24 °C).

The *E. coli* cells were imaged using an inverted fluorescence microscope (IX83, Olympus) with an oil‐immersed objective (Olympus, 150 ×, 1.45 NA) and an EMCCD camera (iXon Ultra 897, Andor). A multi‐wavelength‐combined laser (Cobolt C‐Flex) serves as an excitation light source. Switching among lasers of different wavelengths was achieved by an AOTF (A.A. AOTfnC‐400.605). The laser and the fluorescence were separated by a multi‐band dichromatic mirror and a multi‐band filter. Possible differences in phase and image distortion of different wavelengths were calibrated by four‐color magnetic beads (Thermofisher, TetraSpeck). For TMRM measurements, the indicator (100 nm) was incubated with *E. coli* cells at 37 °C for 60 min to be loaded. The fluorescence of TMRM was excited at 561 nm. In order to obtain images of different excitation wavelengths of pHluorin in one acquisition sequence, the camera and AOTF were controlled with a TTL signal sent by a data acquisitor (National Instrument, USB‐6341) which also recorded the measured voltage applied by a signal generator on the micro‐electrodes.

### In Situ pH Dependence Calibration of pHluorin

In cells expressing pHluorin, 100 µm CCCP and 40 mM potassium benzoate were used to dissipate the pH gradient on the cell membrane, and the cells were fixed on a 0.01% PPL‐modified cover glass, followed by the addition of citrate‐dipotassium hydrogen phosphate buffers of different pH components for 10 min. Bacteria in the center of the frame were selected for statistics and calibration.

### Step‐Photobleaching of pHluorins

Fluorescence time traces were extracted from the fluorescent spots of the immobilized *E. coli* cell continuously excited by a 488 nm laser. The decay curve was filtered by the Chung–Kennedy edge‐holding algorithm to recognize bleaching steps. Pairwise differential distribution power spectrum analysis was used to determine the uniform step size and thus the expected fluorescence of a single pHluorin.

### Proton Diffusion Field Simulation

The proton concentration decreases with the increase of distance from the channel. Regard the channel as a proton points sink,^[^ ^57^ ^]^ and the time‐dependent proton concentration is given by

(1) $$c\left(r,t\right)=c_{\infty }-\frac{q}{2\pi D_{H}r}\left(erfc\left(\frac{r}{2\sqrt{D_{H}t}}\right)\right)$$

where q is the proton flux, D_H_ is the diffusion coefficient of the proton in the cytoplasm, r is the distance from the sink, and t is the elapsed time. In the situation of a stable state, the equation is simplified to

(2) $$c\left(r,t\right)=c_{\infty }-\frac{q}{2\pi D_{H}r}$$

### Data Analysis

Matlab (Mathworks) software was employed for data analysis. Continuous TIRF (Total Internal Reflection Fluorescence) intensity data were processed using the Chung–Kennedy edge‐preserving algorithm,^[^ ^58^ ^]^ which facilitated the determination of individual pHluorin step sizes through Fourier spectral analysis of paired intensity‐difference histograms.^[^ ^59^ ^]^ Intensity correction was performed in accordance with the CoPro method, leading to quantitative single‐channel localization results derived from a single‐molecule localization algorithm (Thunder STROM).^[^ ^60^ ^]^ The resulting photon distribution was converted into the quantified distribution of channels using the fluorescence intensity of a single pHluorin molecule. The quantitative distribution of channels on the bacterial cell membrane was established based on an edge recognition algorithm that extracts bacteria from images, followed by a registration algorithm for superimposed imaging. The time activation constant is extracted by fitting the fluorescence time trace to a single exponential model.

## Conflict of Interest

The authors declare no conflict of interest.

## Author Contributions

J.Z. and Y.X. contributed equally to this work. J.F. conceptualized the project. J.Z., Y.X., J.T. and J.F. developed the methodology. J.Z., S.L. prepared the samples. J.Z., Y.X. conducted the experiments and analyses. J.F., J.T. supervised the work. J.F. and J.T. wrote the manuscript.

## Supporting information



Supporting Information

Supplementary Movie 1

Supplementary Movie 2

Supplementary Movie 3

## Data Availability

The data that support the findings of this study are available from the corresponding author upon reasonable request.
