# Supplementary material for: Spatially Resolved Mapping of Voltage‐Gated Proton Channel Activity Reveals Delayed Proton Transport in Local Microenvironments
Source: Adv Sci (Weinh). 2025 Oct 14;13(2):e10837. doi: 10.1002/advs.202510837 (PMC12786307; doi:10.1002/advs.202510837)
Supplement: Supplementary file 1 — Supporting Information [file ADVS-13-e10837-s002.docx]

Supplementary Information for “Spatially Resolved Mapping of Voltage-Gated Proton Channel Activity Reveals Delayed Proton Transport in Local Microenvironments”

Jiahua Zhuang^1‡^, Yang Xu^1‡^, Yuxian Lu^3^, Shiyang Lyu^1^, Jie Tan^1^* and Jiandong Feng^1,2^*

^1^Laboratory of Experimental Physical Biology, Department of Chemistry, Zhejiang University, 310058 Hangzhou, China

^2^Institute of Fundamental and Transdisciplinary Research, Zhejiang University, 310058 Hangzhou, China

3Hanghzou Institute of Advanced Studies, Zhejiang Normal University, 311231 Hangzhou, China

Table of Contents

[Supplementary Methods 3](#_Toc189322325)

[Supplementary Figures 7](#_Toc189322327)

[Supplementary Movies 1](#_Toc189322328)7

[Supplementary References 1](#_Toc189322330)8

**Supplementary Methods**

**Reagents**

LB (Luria-Bertani medium), M9 (M9 Minimal medium, Shanghai yuanye Bio-Technology Co., Ltd), ampicillin, kanamycin, polylysine, citric acid, K_2_HPO_4_, CCCP (Carbonyl Cyanide m-Chlorophenylhydrazone), TMRM (Tetramethylrhodamine), SDS (Sodium dodecyl sulfate), KCl, NaCl, HEPES (2-[4-(2-hydroxyethyl)piperazin-1-yl]ethanesulfonic acid), β-mercaptoethanol (Macklin), proteinase inhibitors (MedChemexpress), Lysozyme (Macklin), DNAse1 (Shanghai yuanye Bio-Technology Co., Ltd), Ni-NTA agarose (LabLead).

**E. coli strains**

We engineered and constructed a target sequence of pHluorins - the green fluorescent protein ([GFP]) variant at the C-terminal of the Hv1 channel and cloned it onto the pET-28(+) plasmid, which was subsequently transformed into E. coli BL21 (DE3). The transformed cells were oscillated overnight at 220 rpm at 37 °C for pre-culture. And next day the pre-culture was diluted at a ratio of 1:1000 to fresh M9 medium (2% glycerol, 4% amino acids (V/V), 2% Vitamins (V/V)) with 50 μg/mL kanamycin at 37 °C to medium logarithm (OD_600nm_= 0.4-0.5). The expression of Hv1-pHluorins was induced by adding 0.6mM IPTG, and the cells were harvested after continued culture at 37 °C for 4 h for further use.

*Amino acid sequence of Hv1:*

MATWDEKAVTRRAKVAPAERMSKFLRHFTVVGDDYHAWNINYKKWENEEEEEEEEQPPPTPVSGEEGRAAAPDVAPAPGPAPRAPLDFRGMLRKLFSSHRFQVIIICLVVLDALLVLAELILDLKIIQPDKNNYAAMVFHYMSITILVFFMMEIIFKLFVFRLEFFHHKFEILDAVVVVVSFILDIVLLFQEHQFEALGLLILLRLWRVARIINGIIISVKTRSERQLLRLKQMNVQLAAKIQHLEFSCSEKEQEIERLNKLLRQHGLLGEVN

*Amino acid sequence of P2A linker:* GSGATNFSLLKQAGDVEENPGP

*Amino acid sequence of pHluorin:*

MSKGEELFTGVVPILVELDGDVNGHKFSVSGEGEGDATYGKLTLKFICTTGKLPVPWPTLVTTFSYGVQCFSRYPDHMKRHDFFKSAMPEGYVQERTIFFKDDGNYKTRAEVKFEGDTLVNRIELKGIDFKDDGNILGHKLEYNYNEHLVYIMADKQKNGTKAIFQVHHNIEDGGVQLADHYQQNTPIGDGPVLLPDNHYLHTQSALSKDPNEKRDHMVLLEFVTAAGITHGMDELYK

**Purification of Hv1-pHluorins**

The gene of His-tagged Hv1-pHluorins was inserted into the pET-28a(+) plasmid vector, and the recombinant plasmid was loaded in BL21 (DE3) E.coli^1^. The process was done by GenScript Biotech Corp. The transformed cells were grown in 1 L, lysogenic broth medium (LB medium) with 50 μg/mL kanamycin at 37 °C to an OD 600 of 0.6. Expression of Hv1-pHluorins was induced by adding 0.8 mM IPTG and continued growing at 20 °C overnight. Cells were harvested by centrifugation (4500 × g, 30 min, 4 °C). The cell pellets were resuspended in 20 mM HEPES, 150 mM NaCl, 5 mM β-mercaptoethanol, pH 8, and a protease inhibitor cocktail. Lysozyme (320 ug/mL) and DNAse1 (60 μg/mL in 1 M MgCl_2_) were added and the suspension was stirred at 4 °C for 60-90 min. An ultrasonic cell disruptor was used to break the sample for about 50 min (2 seconds ultrasound followed by 2 seconds stop). The broken pieces were separated by centrifugation (4500 × g, 20 min, 4 °C) and the supernatant followed by ultracentrifugation (90000 × g, 4 °C, 90 min). The resulting membrane precipitation was resuspended in 20 mM HEPES, 150 mM NaCl, 1 % SDS (w/v), and pH 8 at room temperature. DNAse1 (2.4 mg) was added and stirred for 60 min followed by centrifugation (15000 × g, 20 °C, 60 min). The remaining supernatant was incubated with Ni-NTA agarose at room temperature overnight. After the resin was rinsed with 3 column volumes of 20 mM HEPES, 150 mM NaCl, 1 % SDS, pH 8, the protein was eluted with 20 mM HEPES, 150 mM NaCl, 1 % SDS (w/v), 250 mM imidazole, pH 8. An SDS-PAGE (sodium dodecyl sulfate-polyacrylamide gel electrophoresis) documented the purity.

**Micro-electrode**

Glass coverslips (0.17 mm thickness, 25 × 25 mm) were cleaned with acetone, isopropanol, and water before photolithography and magnet sputtering. The micro-electrode layer consists of 20 nm Ti and 300 nm Pt. The pattern includes three work electrodes for applying an electric field and one counter electrode that serves as ground. The interval between the work electrode and counter electrode varies from 20 μm to 50 μm as a balance between electric field strength and experiment throughput.

**Cell electrophysiological set-up**

The device consists of a base, a customized PCB (Printed Circuit Board), and a solution chamber. The PCB board is mounted on the base, connecting micro-electrodes through metal clips and signal generator through coaxial interface. The solution chamber is mounted on the PCB board to hold buffers and mediums for cell culture.

**Fluorescence imaging of live E. coli cells**

E. coli cells cultured in 37 °C M9 medium were harvested by centrifugation. The harvested cells were fixed on a 0.01% PLL (Poly-L-Lysine)-modified cover slide, and the unfixed cells were washed away sufficiently by M9 medium. All experiments were conducted at room temperature (22-24 °C).

The E. coli cells were imaged using an inverted fluorescence microscope (IX83, Olympus) with an oil-immersed objective (Olympus, 150×, 1.45 NA) and an EMCCD camera (iXon Ultra 897, Andor). A multi-wavelength-combined laser (Cobolt C-Flex) serves as an excitation light source. Switching among lasers of different wavelengths was achieved by an AOTF (A.A. AOTfnC-400.605). The laser and the fluorescence were separated by a multi-band dichromatic mirror and a multi-band filter. Possible differences in phase and image distortion of different wavelengths were calibrated by four-color magnetic beads (Thermofisher, TetraSpeck). For TMRM measurements, the indicator (100 nM) was incubated with E. coli cells at 37 °C for 60 minutes to be loaded. The fluorescence of TMRM was excited at 561 nm. In order to obtain images of different excitation wavelengths of pHluorin in one acquisition sequence, the camera and AOTF were controlled with a TTL signal sent by a data acquisitor (National Instrument, USB-6341) which also recorded the measured voltage applied by a signal generator on the micro-electrodes.

**In situ pH dependence calibration of pHluorin**

In cells expressing pHluorin, 100 μM CCCP and 40 mM potassium benzoate were used to dissipate the pH gradient on the cell membrane, and the cells were fixed on a 0.01% PPL-modified cover glass, followed by the addition of citrate-dipotassium hydrogen phosphate buffers of different pH components for 10 minutes. Bacteria in the center of the frame were selected for statistics and calibration.

**Step-photobleaching of pHluorins**

Fluorescence time traces were extracted from the fluorescent spots of the immobilized E. coli cell continuously excited by a 488 nm laser. The decay curve was filtered by the Chung-Kennedy edge-holding algorithm to recognize bleaching steps. Pairwise differential distribution power spectrum analysis is used to determine the uniform step size and thus the expected fluorescence of a single pHluorin.

**Proton diffusion field simulation**

The proton concentration decreases with the increase of distance from the channel. Regard the channel as a proton points sink^2^, and the time-dependent proton concentration is given by

c(r,t)=c_∞_$-\frac{q}{2D_{H}r}\left( erfc\left( \frac{r}{2\sqrt{D_{H}t}} \right) \right)$ (1)

where q is the proton flux, DH is the diffusion coefficient of proton in water, r is the distance from the sink, and t is the elapsed time. In the situation of a stable state, the equation is simplified to

c(r,t)=c_∞_$-\frac{q}{2D_{H}r}$ (2)

**Data Analysis**

Matlab (Mathworks) software was employed for data analysis. Continuous TIRF (Total Internal Reflection Fluorescence) intensity data were processed using the Chung-Kennedy edge-preserving algorithm^3^, which facilitated the determination of individual pHluorin step sizes through Fourier spectral analysis of paired intensity-difference histograms^4^. Intensity correction was performed in accordance with the CoPro method, leading to quantitative single-channel localization results derived from a single molecule localization algorithm (Thunder STROM)^5^. The resulting photon distribution was converted into the quantified distribution of channels using the fluorescence intensity of a single pHluorin molecule. The quantitative distribution of channels on the bacterial cell membrane was established based on an edge recognition algorithm that extracts bacteria from images, followed by a registration algorithm for superimposed imaging. The time activation constant is extracted by fitting the fluorescence time trace to a single exponential model.

**Supplementary Figures**


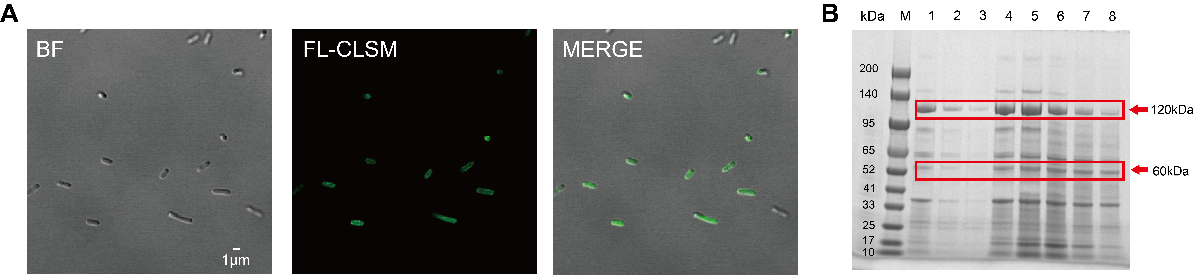


**Figure S1. pH sensitivity and labeling specificity of Hv1-pHluorins.** (A) Localization of Hv1-pHluorin in fixed E. coli cells. The Hv1-pHluorin fusion protein was expressed in E. coli cells. Bright-field (left), corresponding confocal fluorescence scanning microscopy (middle), and the merged (right) image of Hv1-pHluorin localization on the cell membrane of E. coli cells. (B) SDS-PAGE result of purification of Hv1-pHluorins. Representative SDS-PAGE runs with purified Hv1-pHluorins. Lane 1-3: the different fractions 1-3 (F1-F3) obtained in chronological order after affinity chromatography purificationin in 20 mM HEPES, 150 mM NaCl, 1% SDS, 250 mM imidazole, pH 8. Lane 4-8: the different fractions 4-8 (F4-F8) obtained in chronological order after affinity chromatography purificationin in 20 mM HEPES, 150 mM NaCl, 1% SDS, 50 mM imidazole, pH 8. The bands observable at the height of the marker band at 52 to 65 kDa were Hv1s with pHluorins fused to its C-terminus. Another band in the range of 95 to 140 kDa was Hv1-pHluorins dimers.


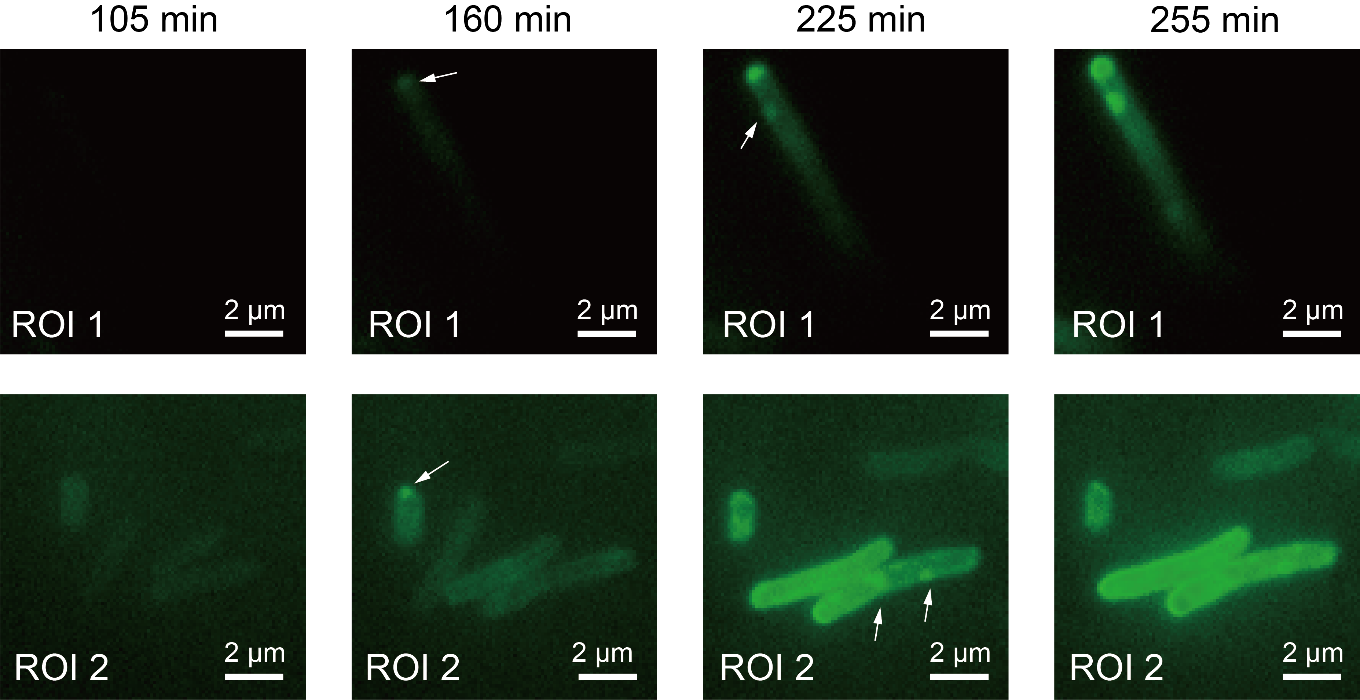


**Figure S2.** **Fluorescence images of E. coli expressing Hv1-pHluorins.** During incubation, the fluorescence intensity of E. coli gradually increased as a result of Hv1-pHluorin expression. Visible fluorescence spots first appear on the edge of E. Coli cells, indicating the successful insertion of Hv1-pHluorin on the inner membrane of e.coli. with prolonged incubation, fluorescence intensity at other sites in E. coli cells also increased, but no sign of inclusion body was observed (aggregated fluorescence spots in e. coli), exclusion the influence of overexpression on the experiment results of our research.


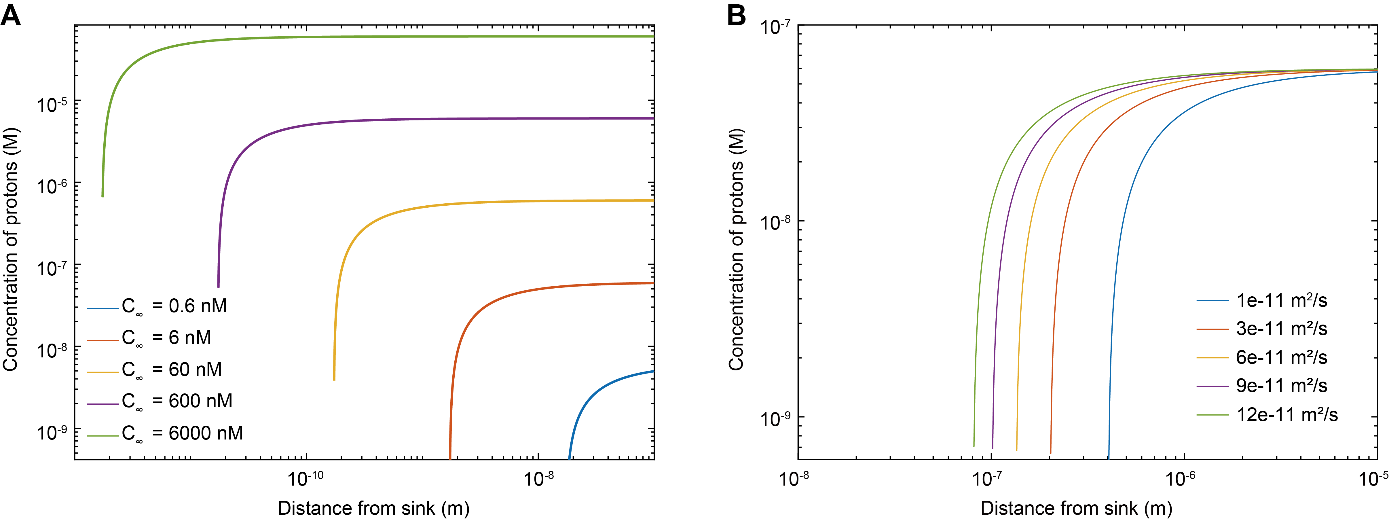


**Figure S3.** **Simulation of the distribution of protons under different bulk proton concentrations.** (A) As described in ***Methods***, proton concentration as a function of distance from the channel was calculated using ***Equation 1***. D_H_ = 7,000 µm^2^/s was used in all curves. In this experiment, c_∞_ = 60 nM; (B) Simulation of proton depletion near the Hv1 channel under different proton diffusion coefficient.


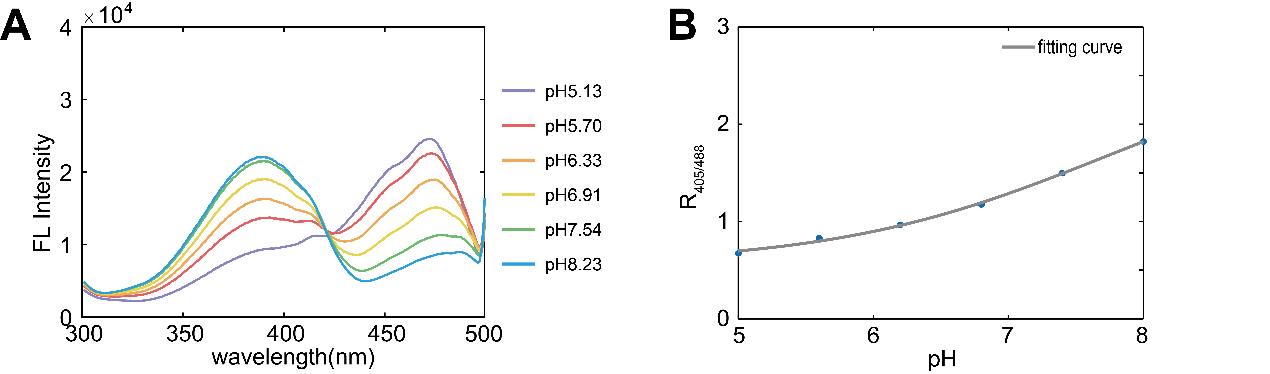


**Figure S4.** **Fluorescence excitation and emission spectra of pHluorin**. The excitation spectra of pHluorin at an emission wavelength of 510 nm and the corresponding ratio. Samples were processed by 100 mM CCCP and then resuspended in phosphate-citrate buffers of different pH.


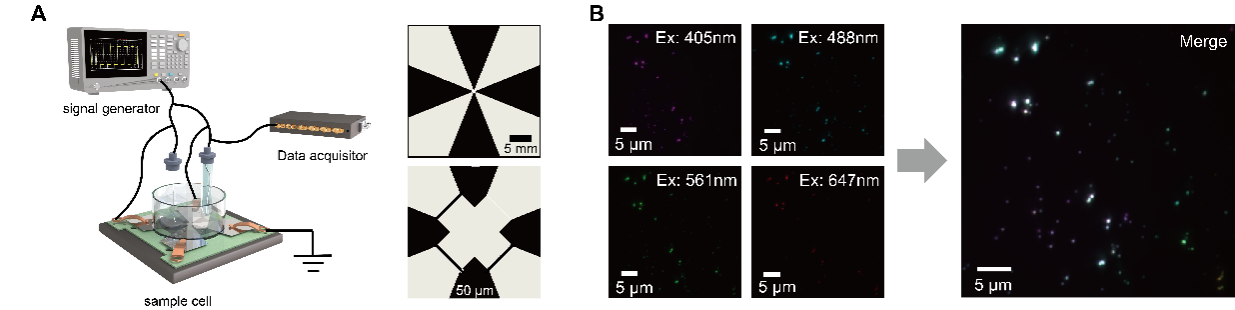


**Figure S5.** **Cell electrophysiological set-up and data acquisition system for Hv1.** (A) Left: as described in ***Methods***, the cell electrophysiological set-up consists of a base, a PCB board, and a solution chamber that is compatible with glass electrodes. The patterned electrode and two glass electrodes consist of a three-electrode system for accurate voltage application and recording. Right: the patterned electrode and zoomed image. (B) Left: the four-color micro-beads image excited at 405 nm, 488 nm, 561 nm and 647 nm. Right: the multi-channel merge image. The results demonstrated that the system exhibits excellent imaging performance when transitioning between 405 nm and 488 nm wavelengths, and is suitable for ratiometric fluorescence analysis without introducing artifacts.


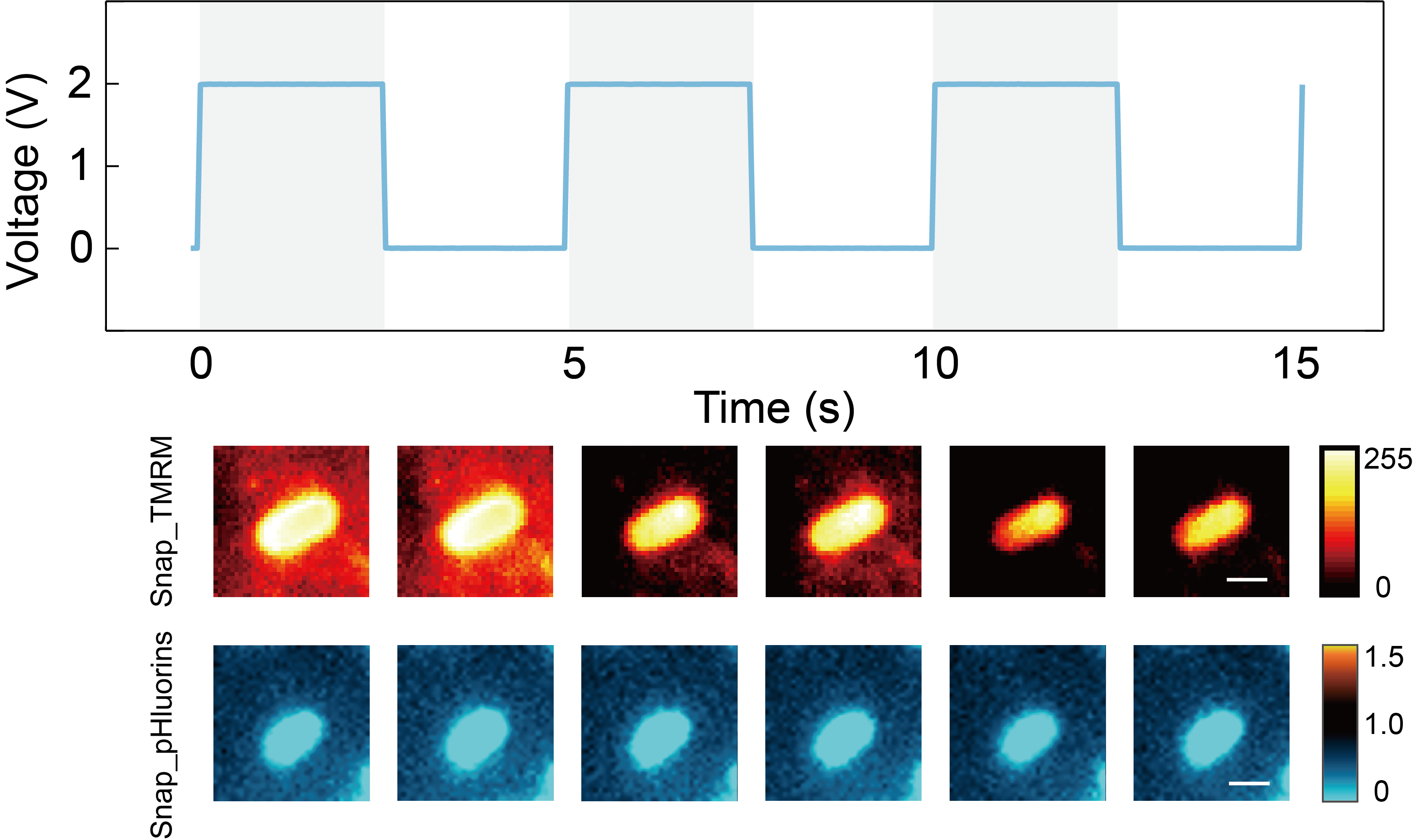


**Figure S6.** **The fluorescence images corresponding to the traces in *Fig 2B*.** Top, the voltage applied on micro-electrodes. Bottom, snaps of bacteria (TMRM/pHluorins). Unlike ratiometric Hv1-pHluorins probe, bare pHluorins reflect weak response to voltage stimulation.


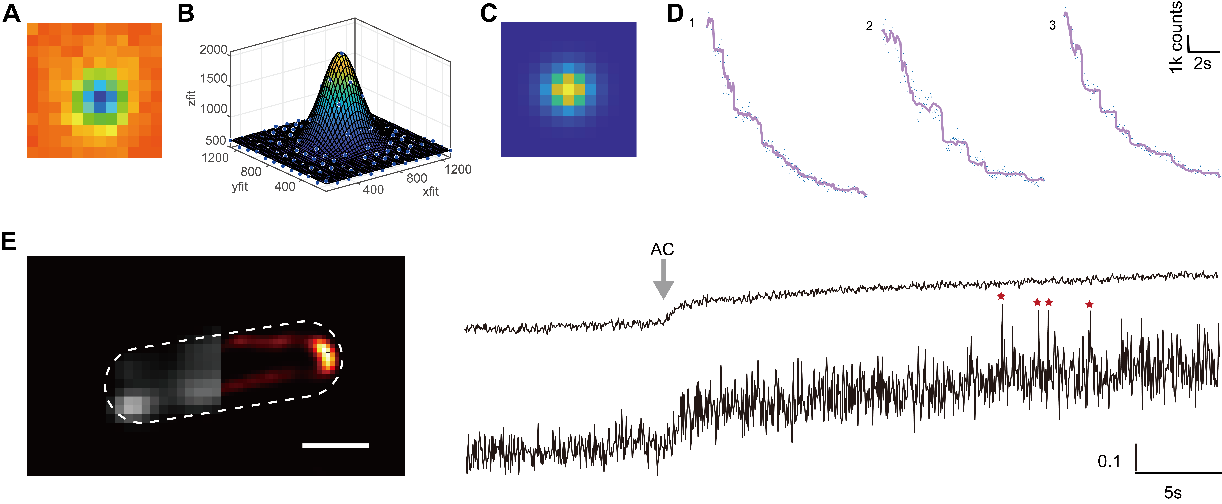


**Figure S7.** **Observation of single channels**. (A) The raw image obtained using 100 nm microbeads. (B) The original data fitted by 2D Gaussian. (C) The extracted point spread function of our system. (D) Time traces of different fluorescence spots show parallel photobleaching traces (blue dots). Chung-Kennedy-filtered traces show distinct stepwise bleaching behavior (purple curves). (E) When comparing the ratio time trace of whole-cell (upper curve) to that of a single-channel (lower curve) after applying an external electric field, we found spike signals (≥ 3 × std., marked with red stars) in the time trace of single-channel. These findings may suggest certain peculiarities of single-channel behavior. By employing detectors with higher sampling rates, further investigations are possible.


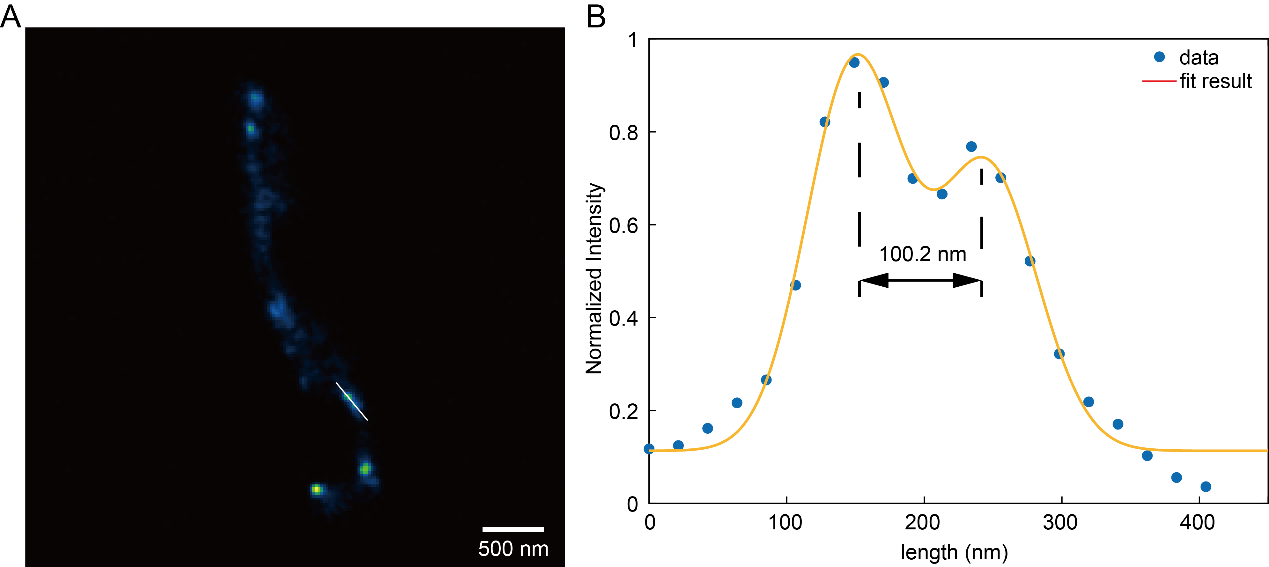


**Figure S8.** **Spatial resolution analysis**. (A) Selected region from ***Fig. 3F***; (B) Intensity profile according to the white line in A. The achieved resolution is 100.2 nm.


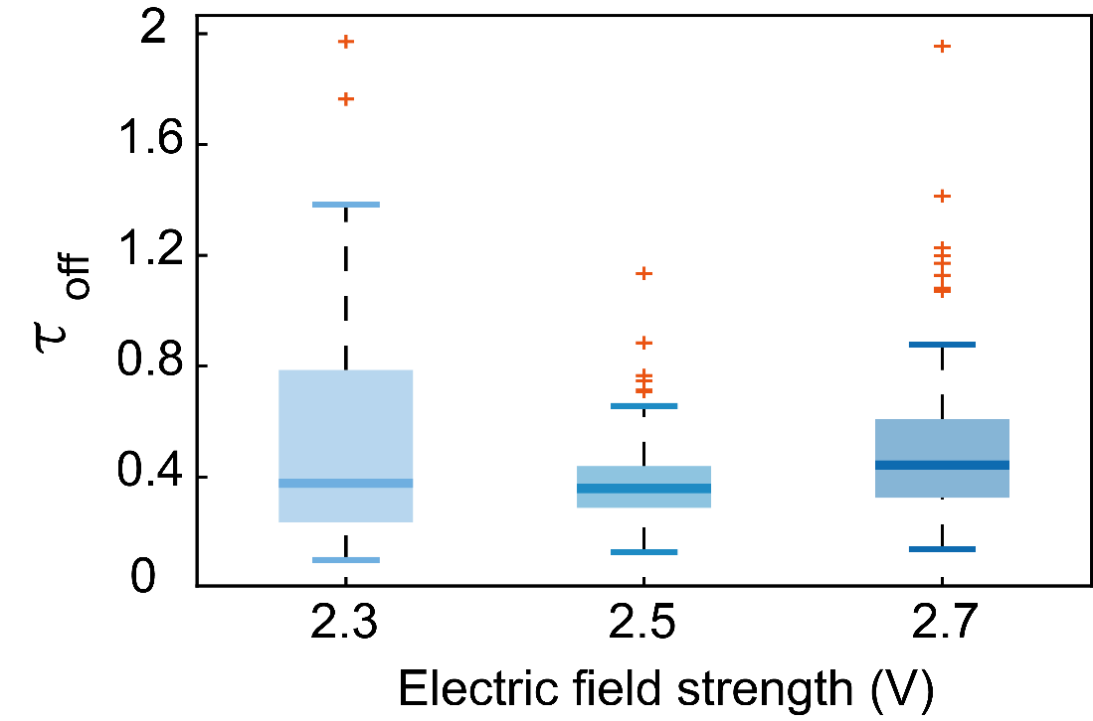


**Figure S9.** **The statistic result of deactivation time constants at different electric field strengths**. The ***τ****_off_* at different electric field strengths is 0.377, 0.358, 0.443 s in turn. From the boxplot above, there are no appreciable alteration in the deactivation time constants comparing to the activation time (***Fig. 4F***). The p-values for ***τ****_off_* data of 2.3 V and 2.5V, data of 2.3 V and 2.7 V and data of 2.5 V and 2.7 V are 0.0044,0.23, and 0.0022 respectively.


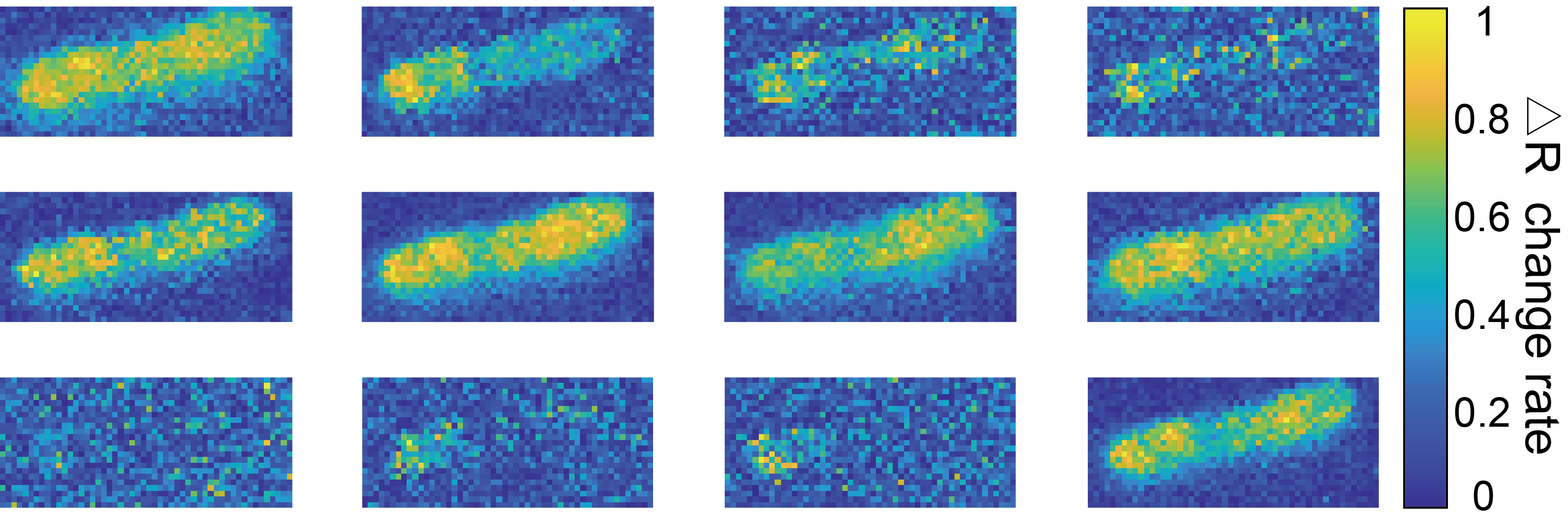


**Figure S10. Spatial-temporal analysis of electric field regulated Hv1-pHluorin behaviors in single E. coli cell.** The corresponding pH change of each image in the ***Fig. 5C***. As expected, it is readily observable that the maxima of fluorescence ratio change coincide with the localizations of Hv1-pHluorins. However, from the pH change mapping result, it is clearly that the fluorescence ratio of Hv1-pHluorin with highest activity continues to change after the fluorescence ratio of other Hv1-pHluorins reaches equilibrium state. The result indicated that local proton concentration of Hv1 channels with higher activity is higher than those of lower activity.


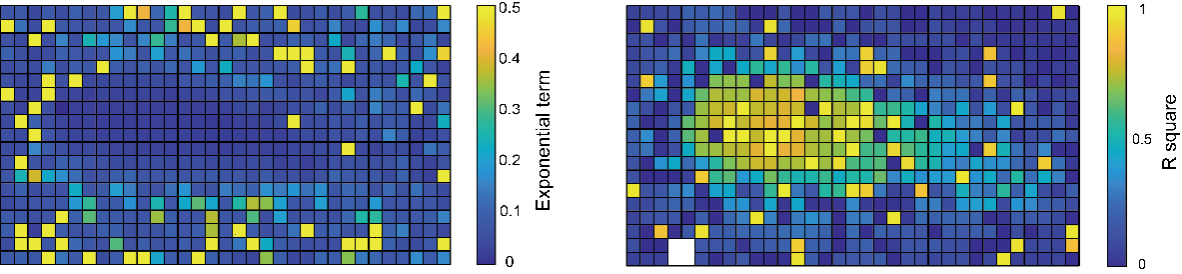


**Figure S11. The mapping of fitted decaying rate of single E. coli and the corresponding *R^2^* value.** The exponential term is higher near the edge of the E. coli cell than in the middle, indicating a higher decaying rate near the edge, which conforms with the fact that Hv1-pHluorin is a membrane protein. Correlating with the *R^2^* value mapping, we confirm the mapping result is reliable.


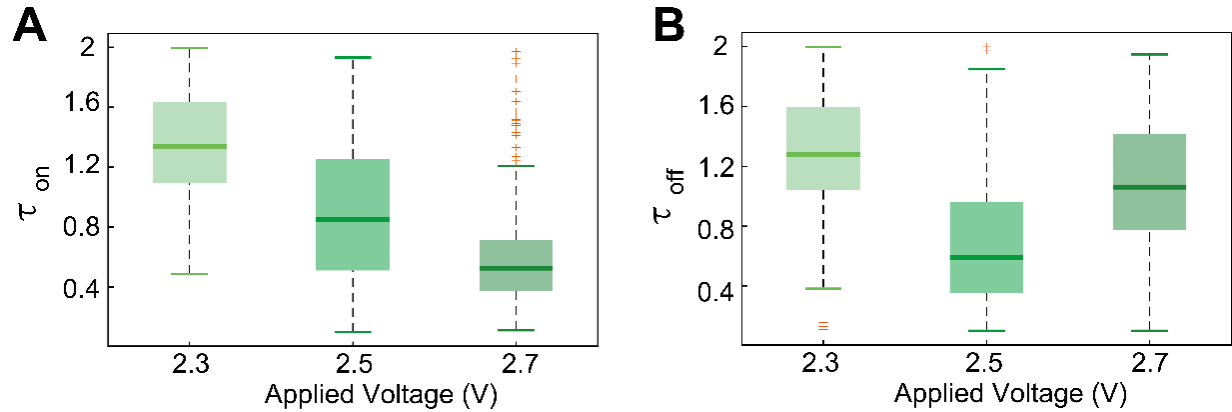


**Figure S12.** **The statistic result of activation time constants and deactivation time constants at different electric field strengths in Hypertonic solution environment**. (A) The statistic result of activation time constants in hypertonic solution suggests same tendency as observed in physiological conditions (***Fig.6B***) that the activation time constant decreases with the increase of electric field strength. The P-values for t_on_ data of 2.3 V and 2.5V, data of 2.3 V and 2.7 V and data of 2.5 V and 2.7 V are 4.2×10^-6^,6.4×10^-10^, and 2.0×10^-9^ respectively. (B) The statistic result of deactivation time constants in hypertonic solution shows no distinct pattern with the change of electric field strength. The p-values for t_off_ data of 2.3 V and 2.5V, data of 2.3 V and 2.7 V and data of 2.5 V and 2.7 V are 6.4×10^-6^,0.11, and 6.4×10^-10^ respectively.

**Supplementary Movies**

**Movie S1. Ratiometric fluorescence change of Hv1-pHluorins in E. coli under pulsed voltage stimulation.** A square wave signal (0-2.3 V, 0.2 Hz) was continuously applied on a pair of micro-electrodes. The fluorescence ratio of Hv1-pHluorin changes in response to the change of voltage stimuli. The buffer used in this experiment is 100 mM HEPES, pH 7.5. Scale bar: 1 μm.

**Movie S2.** **The stepwise photobleaching video of a Hv1-pHluorin E.coli cell.** The E. coli cell was illuminated continuously with 488 nm laser (~1.70 mW). Exposure time was set to 50 ms. The buffer used in this experiment is 100 mM HEPES, pH 7.5.

**Movie S3. Ratiometric fluorescence change of Hv1-pHluorins in E. coli under pulsed voltage stimulation in a hypertonic environment.** A square wave signal (0-2.3 V, 0.2 Hz) was continuously applied on a pair of micro-electrodes. The fluorescence ratio of Hv1-pHluorin changes in response to the change of voltage stimuli. The buffer used in this experiment is 100 mM HEPES, pH 7.5 containing 20% sucrose. Scale bar: 2 μm.

**Supplementary Reference**

(1) Gerdes, B.; Rixen, R. M.; Kramer, K.; Forbrig, E.; Hildebrandt, P.; Steinem, C. Quantification of Hv1-Induced Proton Translocation by a Lipid-Coupled Oregon Green 488-Based Assay. *Anal. Bioanal. Chem.* **2018**, *410* (25), 6497–6505.

(2) De-la-Rosa, V.; Suárez-Delgado, E.; Rangel-Yescas, G. E.; Islas, L. D. Currents through Hv1 Channels Deplete Protons in Their Vicinity. *J. Gen. Physiol.* **2016**, *147* (2), 127–136.

(3) Chung, S. H.; Kennedy, R. A. Forward-Backward Non-Linear Filtering Technique for Extracting Small Biological Signals from Noise. *J. Neurosci. Methods* **1991**, *40* (1), 71–86.

(4) Svoboda, K.; Schmidt, C. F.; Schnapp, B. J.; Block, S. M. Direct Observation of Kinesin Stepping by Optical Trapping Interferometry. *Nature* **1993**, *365* (6448), 721–727.

(5) Ovesný, M.; Křížek, P.; Borkovec, J.; Švindrych, Z.; Hagen, G. M. ThunderSTORM: A Comprehensive ImageJ Plug-in for PALM and STORM Data Analysis and Super-Resolution Imaging. *Bioinformatics* **2014**, *30* (16), 2389–2390.
